# miR-34 regulates stress-induced depression-like state through the *WDR26* ortholog *melancholy* in *Drosophila*

**DOI:** 10.21203/rs.3.rs-9339604/v1

**Published:** 2026-04-08

**Authors:** Shreyasi Mitra, Amit Kumar, Aman Gill, Majid Ali, Aman Bashar, Puli Chandramouli Reddy, Geetanjali Chawla

**Affiliations:** 1 RNA Biology Laboratory, Department of Life Sciences, School of Natural Sciences, Shiv Nadar Institution of Eminence, Uttar Pradesh-201314, India; 2 CSIR-Institute of Genomics and Integrative Biology, Council of Scientific and Industrial Research (CSIR), Delhi-110025, India; 3 Center of Excellence in Epigenetics, Department of Life Sciences, School of Natural Sciences, Shiv Nadar Institution of Eminence, Uttar Pradesh-201314, India

**Keywords:** MicroRNAs, depression, miR-34, stress, behaviour

## Abstract

Major depressive disorder (MDD) is a complex and recurrent neuropsychiatric disorder with poorly understood molecular underpinnings. Psychosocial stress is a major environmental risk factor, yet the mechanisms linking stress exposure to behavioral outcomes remain unclear. MicroRNAs (miRNAs) have emerged as key regulators linking environmental stress to changes in gene expression in mood disorders. Here, using stress-induced rodent and *Drosophila* models, we identify the conserved, brain-enriched miRNA, miR-34, as a critical mediator of stress responses. Our expression analysis revealed significant downregulation of miR-34 in brain tissues from stress-induced depressed rodent and fruit fly models. We showed that this stress-induced downregulation of miR-34 is due to the upregulation of its transcriptional inhibitor *Broad*. Loss of miR-34 in flies induces depression-like behavior even in the absence of stress, whereas its overexpression in serotonin-sensing neurons confers resilience. We identify a conserved regulatory axis between miR-34 and *CG7611*, encoding the *WDR26* ortholog *Melancholy* (*Mel*). Reducing *mel* levels mimics miR-34 overexpression, while its upregulation induces depression-like phenotypes. Proteomic analysis shows that *mel* overexpression recapitulates a stress-like state, characterized by downregulation of cytoskeletal, nuclear pore, chromatin, and transcriptional proteins, alongside upregulation of mitochondrial, metabolic, and detoxification pathways. These coordinated changes indicate a shift toward a low-activity, neuroprotective neuronal state driven by nuclear reprogramming and metabolic adaptation. Together, our findings reveal a conserved, adult-specific role for the CTLH complex in stress responses and establish miR-34 as a dosage-sensitive regulator of depression-like states.

## INTRODUCTION

Major depressive disorder (MDD) is a multifactorial, heterogeneous, and complex mood disorder that is influenced by genetic, environmental, and epigenetic factors(1). MDD is characterized by symptoms of persistent anhedonia (loss of interest or pleasure in daily activities), feelings of low esteem/guilt, disrupted sleep patterns, loss of appetite, fatigue, poor concentration, impaired cognitive function, and suicidal thoughts(2). Some reported causal mechanisms of MDD include dysregulation of neuronal transmission systems (serotonin, dopamine, norepinephrine, glutamatergic, and GABAergic transmission), glial cell function, blood-brain barrier integrity, structural brain changes, and inflammation (3). However, none of these mechanisms alone can fully explain the different pathological manifestations of MDD. Epidemiological studies indicate that environmental factors such as chronic stress are associated with the risk of developing MDD, yet all individuals who experience stress donot develop this disorder(4-7). Several animal models have been used to model stress-induced depression phenotypes and to identify resilience-associated genes. Various chronic unpredictable mild stress (CUMS)-induced models have opened the possibility of examining sex-specific responses to stress(4).

MicroRNAs (MiRNAs) are a class of small noncoding RNAs that function as dosage-sensitive post-transcriptional regulators of gene expression(8). They are approximately 21-24 nt long and regulate gene expression by binding to their target RNAs via imperfect base-pair interactions. These interactions lead to the silencing of the target RNA, either through translational inhibition or degradation of the miRNA targets (9). Several studies have reported alteration of miRNAs in response to stress and an association between microRNA levels and the onset and progression of neuropsychiatric disorders, including MDD(10-12). MiRNAs play a regulatory role in the MDD brain by altering the gene expression landscape, which in turn drives changes in synaptic plasticity, neurogenesis, and hypothalamic-pituitary axis function. They act as an epigenetic interface, facilitating the rapid, large-scale changes in cellular pathology in response to environmental stimuli in a highly coordinated manner. Compartmentalization of miRNAs, variation in their localization, and changes in their expression levels lead to significant perturbations in the MDD brain. The conserved microRNAs let-7, miR-124, miR-34, miR-16, miR-17, miR-18, miR-30, miR-29, miR-139, miR-145, miR-193, miR-203, miR-192, miR-215, miR-219, and miR-338 have been found to change in response to stress-induced depressive conditions in humans and animal models(10,13,14).

A critical gap that remains in the field is understanding how specific stress-responsive miRNAs mechanistically contribute to depression-like behavior by serving as independent risk factors, and whether their expression levels exert dosage-dependent effects on neural circuitry and concomitantly the observed symptoms. Although many miRNAs have been implicated in MDD, only a small subset has been causally linked to behavioral outcomes, and in most cases, the downstream molecular pathways remain poorly defined. Despite the recognized importance of stress-responsive miRNAs, their functional outcomes are often profoundly dosage-dependent. Small changes in miRNA expression can lead to disproportionate shifts in neuronal circuit activity, altering behavioral responses toward resilience or vulnerability. However, the behavioral consequences of precise and localized changes in miRNA dosage during psychosocial stress remain poorly understood.

Currently, the most commonly prescribed treatments include Selective serotonin reuptake inhibitors (SSRI) and serotonin-norepinephrine reuptake inhibitors (SNRI) administration, which have high remission rates and several deleterious side effects(15,16). Some commonly used SSRIs include Fluoxetine, escitalopram, benzodiazepines, and selective norepinephrine reuptake inhibitors (SNRIs). Additionally, growing evidence suggests that several antidepressants exert their effects by targeting miRNAs(17-19). Therapeutic approaches, such as Cognitive Behavioral Therapy (CBT) and Electroconvulsive Therapy (ECT), have also been shown to alter miRNA expression levels(20,21). These studies highlight the role of miRNAs as diagnostic biomarkers and in predicting the response to therapeutic approaches.

Many stress-responsive miRNAs are deeply conserved across bilaterians, suggesting that fundamental miRNA-mediated regulatory mechanisms governing behavioral adaptation arose early in evolution. *Drosophila* shares most ancient miRNA families with mammals, including miR-34, miR-124, miR-16, let-7, and others whose expression changes in response to stress-induced depressive states in rodents and humans. Among conserved neuronal miRNAs, miR-34 stands out as a particularly intriguing candidate. Most studies in vertebrates indicate that the miR-34 family is robustly induced by chronic stress and has been associated with anxiety, depressive symptoms, cognitive impairment, and stress-induced structural plasticity(22-25). In contrast, other studies have identified the downregulation of miR-34 family members (miR-34a and miR-34c) in contexts related to depression, particularly in the brain tissues and specific stress models (14,26). Downregulation of miR-34 family members has also been reported in motor neuron degeneration, following spinal cord injury, neurotoxic stress models, and latestage Alzheimer's disease models(27-29). Unlike mammals, *Drosophila* has one miR-34 locus. This miRNA is maternally inherited, highly expressed in the nervous system, and its expression increases with age (30,31). *Drosophila* miR-34 regulates neuronal maintenance, innate immunity, aging, synaptic robustness, and neuroprotection, and its expression increases in response to multiple physiological stressors. (32-34). Moreover, miR-34 has been shown to exhibit heterogeneity in length and sequence across different cell types and biological contexts (35). Despite these studies, its causal role in stress-induced depression-like behavior and the significance of its dosage in modulating affective outcomes remain unexplored. *Drosophila* provides an exceptional genetic model to address this gap. Flies exhibit quantifiable depression-like phenotypes—including anhedonia, learned helplessness, reduced motivation, and behavioral despair—that are induced by psychosocial stress paradigms such as social defeat or repeated social threat. Many molecular pathways regulating stress response and mood—such as monoaminergic signaling, neuropeptide systems, immune pathways, and chromatin regulators—are conserved between flies and mammals. The ability to manipulate miRNA levels using precise genetic tools makes *Drosophila* a uniquely powerful system for dissecting dosage-sensitive miRNA–target interactions that drive behavioral outcomes.

In this study, we set out to identify and characterize miRNA-mediated mechanisms in depression-like behavior. We established a stress regimen to induce a depression-like state in Long-Evans rats and *Drosophila* melanogaster and identified that miR-34 was downregulated in the hippocampal tissue of stressed rats and in the head tissue of stressed flies. The stressed rodent and fly models displayed behavioral deficits that were analogous to the symptoms of major depressive disorder. Using molecular, genetic, and proteomic approaches, we identified a new miR-34-melancholy axis that regulates stress-induced depression like behavior. Our analysis reveals the potential utility of modulating miR-34 or its target, *melancholy*, as a therapeutic and diagnostic strategy for depression.

## RESULTS

### Chronic Unpredictable Mild Stress (CUMS) induces robust behavioral dysfunction and downregulation of miR-34 expression in Long-Evans Rats

**Fig. 1.**
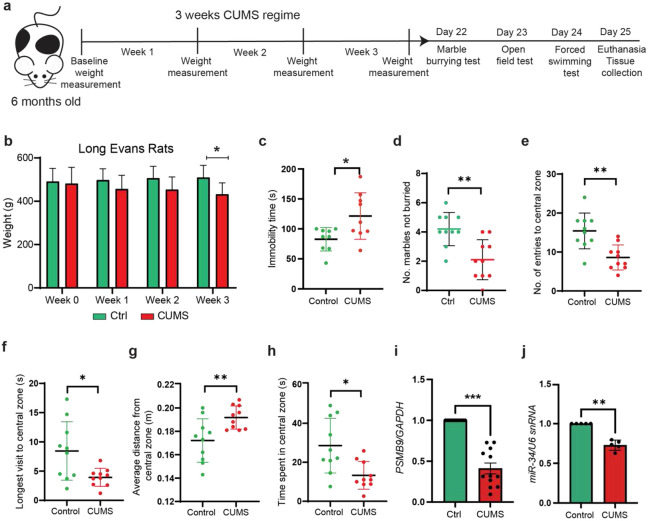
Chronic unpredictable mild stress (CUMS) induces behavioral phenotypes and miR-34 downregulation in rats. **(a)** Schematic of the 3-week CUMS paradigm applied to 6-month-old rats, including weekly weight measurements and subsequent behavioral testing: marble burying (day 22), open field (day 23), forced swimming (day 24), and tissue collection (day 25). **(b)** Body weight assessment in control and CUMS-exposed Long-Evans rats over the 3-week period indicates that the body weight of the control and CUMS groups shows differential progression during the 3-week CUMS regimen, and CUMS rats weigh significantly less than control rats at week 3. **(c)** The CUMS-exposed Long-Evans rats displayed a significantly increased immobility time. **(d)** The number of marbles not buried in the marble burying test indicates greater compulsive behavior in CUMS rats in comparison to their controls. **(d)** Immobility time in the forced swimming test is increased in CUMS rats in comparison to control rats, indicating a depression-like state characterized by despair. **(e–h)** Open field test parameters, including the number of entries to the central zone **(e)**, the longest visit to the central zone **(f)**, average distance from the central zone **(g)**, and total time spent in the central zone **(h)**, indicate exacerbated anxiety-like responses in CUMS rats in comparison to their controls. **(i)** *PSMB9* expression is significantly downregulated in the hippocampal tissue of CUMS rats relative to controls. Values are mean ± SD, n=6. For each biological replicate (n=3), two technical replicates were analyzed. **(j)** Hippocampal miR-34 expression levels normalized to *U6 snRNA* in CUMS rats are downregulated in comparison to control rats. Values are mean ± SD, n=5. For each biological replicate (n=5), two technical replicates were analyzed. CUMS exposure resulted in altered anxiety- and depression-like behaviors, including reduced exploratory activity in the central zone, increased immobility, and changes in marble burying performance, along with reduced miR-34 expression. n=10 in Panel C-H, n=12 in Panel A and n=5 in panel I. Data is presented as mean ± S.D. with individual data points shown. Statistical significance was determined using a multiple t-test with FDR correction and Two-way ANOVA in Panel B, a non-parametric Mann-Whitney test in Panels C to J, and an unpaired t test with Welch’s correction in Panel I-J; *P < 0.05, **P <0.01.

To assess the physiological and psychological effects of chronic environmental stress, we subjected 6-month-old Long-Evans rats to a 3-week CUMS regimen (Fig. 1a). We first assessed the physical impact of the stressor, finding that CUMS-treated rats exhibited significantly attenuated weight gain compared to control animals, with a significant reduction in body weight observed by the third week of the protocol (Fig. 1b). Following the stress period, a battery of behavioral assays revealed the induction of a robust anxiety- and despair-like phenotype. A marked increase in marble-burying activity, as evidenced by a lower number of marbles left unburied in the CUMS group (Fig. 1c), indicated a compulsive behavioral state of CUMS rats. Furthermore, stressed rats exhibited a significant increase in immobility time during the Forced Swim Test (FST), a hallmark of behavioral despair (Fig. 1d). In the Open Field Test (OFT) used to assay exploratory behavior in a novel arena, CUMS rats displayed classic thigmotaxic behavior, characterized by a significant reduction in the number of entries to the central zone (Fig. 1e), shorter duration of the longest central visit (Fig. 1f). This increased anxiety was further corroborated by a significant increase in the average distance from the central zone and overall, less time spent in the center of the arena (Fig. 1h-g). To validate whether these stressed animals displayed gene expression changes that have been reported in previously published studies that have utilized CUMS models, we examined the expression of Proteasome subunit-beta type 9 (*PSMB9)*, an immunoproteasome component that is involved in the processing of viral proteins and the Class I peptides of the Major Histocompatibility Complex-I (MHC-I) (36,37). To identify potential differentially expressed miRNA correlates of these behavioral changes, we quantified miR-34 levels in the hippocampus of control and stressed rats, revealing a significant downregulation of miR-34 in stressed animals compared to controls (Fig. 1i). Collectively, these results demonstrated that chronic stress exposure in rats not only drives comprehensive affective dysfunction and physiological weight loss but also significantly suppresses miR-34 expression in the hippocampus, suggesting a potential conserved role for this microRNA in maintaining stress resilience.

### MiR-34 levels are depleted in the brain tissue of psychosocially and pharmacologically stressed wild-type flies

**Fig. 2.**
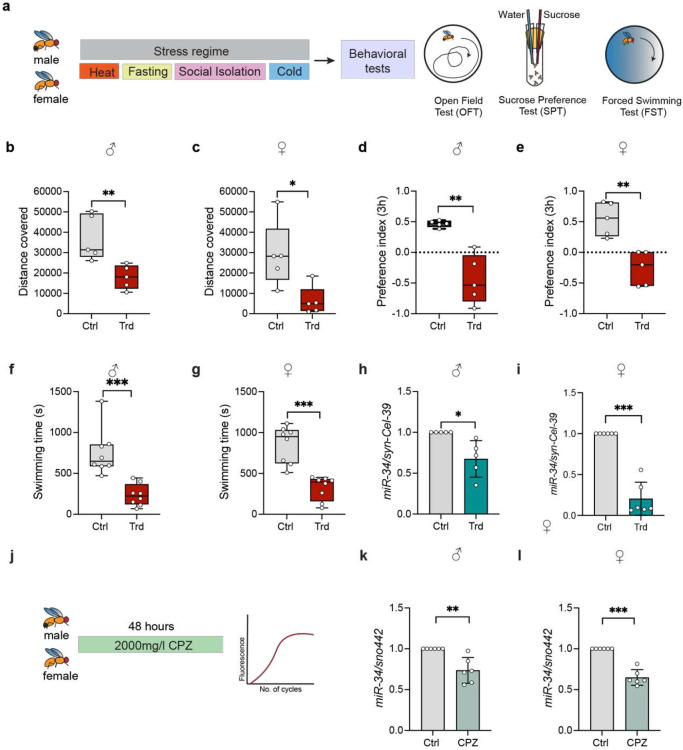
Administration of a stress regime to wild-type *Canton-S* flies leads to exploratory deficits and a reduction of miR-34 in head tissue. **(a)** A schematic diagram representing the short stress regime employed to induce depression-like behavior in 3-5 days old *Drosophila melanogaster*, consisting of heat shock at 37°C (20 minutes for males and 30 minutes for females), fasting (6 hours for males and 8 hours for females), social Isolation for 18 hours and cold shock at −5°C (10 minutes for males and 20 minutes for females). After administration of this regimen, flies were subjected to three behavioral tests: the open field test, the sucrose preference test, and the forced swimming test. **(b-c)** Exploratory behavior of control and stressed male **(b)** and female **(c)** flies. Stressed flies traveled a shorter distance in the open-field arena, indicating anxiety-like behavior. **(d-e)** PI of male **(d)** and female **(e)** *Canton-S* flies. Panel 1 indicates control flies, panel 2 (Trd) indicates stressed flies. 3-5-day-old flies exposed to a stress regimen show a significant reduction in PI. **(f-g)** Forced swim test performance in males **(f)** and females **(g)**, reflecting helplessness-like behavior. Stressed flies displayed a decreased swimming time. **(h-i)** MiR-34 levels were measured in the head tissue of control and stress-exposed male **(h)** or female **(i)** flies, which were subjected to a stress regimen. **(j)** Dietary strategy for Chlorpromazine (CPZ) administration followed by qRT-PCR. **(k-l)** MiR-34 levels were measured in the head tissue of controls and in those administered 2000 mg/l CPZ. MiR-34 levels were downregulated upon CPZ exposure. Statistical analysis for qRT-PCR analysis was done using an unpaired t-test with Welch’s correction. The p-value threshold was set at 0.05 to assess significance. *p<0.05, **p<0.01, ***p<0.005 and ^ns^p>0.05. Genotype of strain used in this figure **(a-l):** *Canton-S*

*Drosophila melanogaster* has emerged as a genetically tractable model for studying the neurobiology of depression-like states, and because flies have a single copy of miR-34, we established a stress regimen to induce depression-like states in *Drosophila* to functionally characterize miR-34's role in depression(31,38)(Fig 2a). *Drosophila* miR-34 is a brain-enriched, adult-onset miRNA known to have regulatory roles in longevity and neuroprotection. MiR-34-knockout flies exhibit decreased survival and large vacuoles in their brain tissue, characteristic of neurodegeneration, along with dramatic climbing defects(30). MiR-34 affects synapse formation and maintains the structural integrity of brain tissue, with a role in the maternal regulation of neural development(31-33). At the molecular level, miR-34 regulates protein homeostasis in flies, modulates the ecdysone signaling pathway, and modulates innate immune responses(34). To characterize the role of miR-34 in depression-like states in *Drosophila*, we used our recently published variable stress protocol, which induces anhedonia and behavioral despair in both male and female wild-type flies, as measured by the sucrose preference test (SPT) and the forced swimming test (FST), respectively(39). To further understand the effects of this regime on exploratory behavior, we employed an Open Field Test (OFT) adapted for flies. This assay is a measure for exploratory behavior in a novel arena, with reduced exploration correlating to anxiety-like behaviors. 3-5-day-old wild-type flies were sorted by sex and exposed to a stress regimen consisting of heat shock at 37°C, fasting, social isolation, and cold shock at −5°C. Flies were then individually transferred to a novel arena, allowed to acclimatize for 2 minutes, and their movement was recorded for 5 minutes. Both male and female flies subjected to a stress regime showed a significant reduction in total distance covered by the fly during the 5 minutes of recording time, indicating an anxiety-like response to stress. Total distance covered reduced from 37170 ±11166 a.u. to 18004 ±5836 a.u. in males and from 29040 ± 16052 a.u. to 6335 ± 7086 a.u. in females upon exposure to stress regime (Fig 2b-c). Another typical symptom of depression is the lack of interest in enjoyment or pleasure, also referred to as Anhedonia(40). To quantify anhedonia, we performed a sucrose preference test (SPT). Control flies and stressed flies were offered a choice between 5% sucrose and water, and their anhedonia was tested through the sucrose preference test (SPT) for 3 hours. Stressed *Canton-S* male and female flies showed a reduced preference for sucrose (Preference Index {PI} dropped from 0.4655 ± 0.05 to −0.4459 ± 0.3986 in males and from 0.5427 ± 0.2739 to −0.2586 ± 0.2752 in females), indicating disinterest in reward-seeking activities (Fig. 2d-e). To further validate depression-like phenotypes in *Drosophila*, we employed a Forced Swimming Test (FST) to quantify behavioral despair by measuring the latency to immobility in an aqueous column, providing an independent index of motivational state and stress-coping behavior(41,42). Both male and female flies that were exposed to a stress regime exhibited a significant reduction in latency to immobility (Immobility time decreased from 753.375 ± 284.98 seconds to 235.625.33 ± 133.235 seconds in males and from 854.375 ± 225.714 to 320.625 ± 147.28 in females) (Fig. 2f-g).

To elucidate whether miR-34 functions in the regulation of psychological stress responses, the levels of miR-34 were measured in the brain tissue of flies exposed to a chronic stress regime, and their expression was compared to that of age-matched controls. MiR-34 levels are depleted in the brain tissue of flies exposed to the stress regime, with a 27.96% decrease in males (Fig. 2h) and 79.59% decrease in females (Fig.2i). We further analyzed whether miR-34 levels are altered upon pharmacological induction of depression using chlorpromazine, a pharmacological intervention to induce depression-like phenotype by acting as a monoamine receptor antagonist(43) (Fig. 2j). Supplementation of fly food with 2000mg/L chlorpromazine led to a 26.48% decrease in miR-34 levels in males and 35.16% decrease in females, indicating that pharmacological stressors also impact miR-34 levels in the brain of wild-type flies (Fig. 2k-l). These data indicate that miR-34 is downregulated by psychosocial stressors and pharmaceutical interventions that induce depression-like behavior.

### MiR-34 is transcriptionally regulated by Broad-Complex under stress conditions

**Fig. 3.**
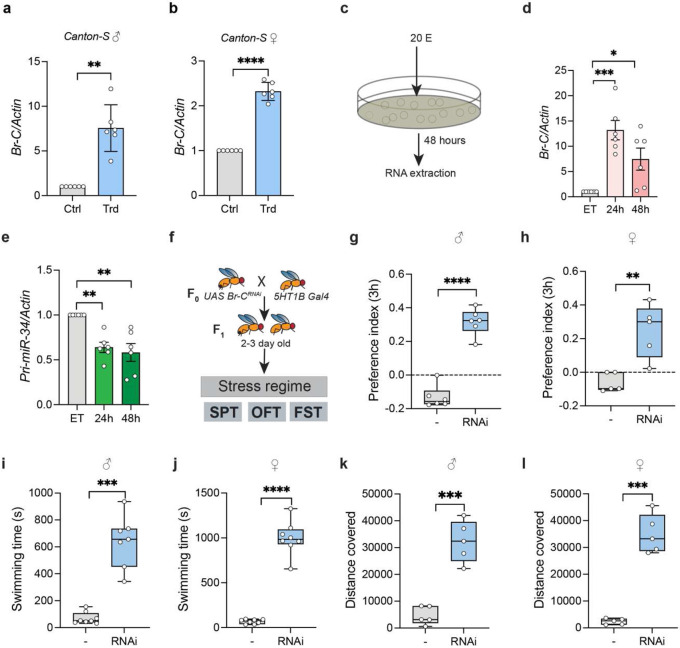
Broad transcriptionally regulates miR-34 under stress conditions and provides relief from stress responses upon knockdown. **(a-b)** RT-PCR was used to quantify Broad mRNA levels in male and female flies, indicating that Broad levels are upregulated in stressed brains of male **(a)** and female **(b)** flies. **(c)** Schematic representation of 20-Hydroxyecdysone (20E) treatment in Kc167 cells followed by RNA extraction. **(d-e)** Broad and primary-miR-34 levels are increased and decreased, respectively, upon administration of 20E to Kc167 cells. **(f)** Schematic representation of the strategy to knock down Broad in 5-HT1B neurons. **(g-l)** Knockdown of Broad leads to significant relief from stress-induced depression-like behavior in male and female flies, exemplified by an increase in sucrose preference, indicating a decrease in anhedonia **(g-h)**, increased swimming capabilities in male and female flies **(i-j)**, and robust exploratory behavior shown by males and females in the open field **(k-l)**. Statistical significance was determined using an unpaired T-test with Welch’s correction. P-value threshold was set at 0.05. *p<0.05, **p<0.01,***p<0.005, **-=]**p<0.001. Genotype of strains used in this figure **(g-l)** *5HT1B Gal4> UAS Br*^*RNAi*^: *+/+;P{w[+mC]=5-HT1B-GAL4.Y}3 /y[1] v[1]; P{y[+t7.7] v[+t1.8]=TRiP.HMS00042}attP2/TM3, Sb[1]*.

The regulation of miR-34 in *Drosophila* is a tightly controlled transcriptional process driven by the steroid hormone ecdysone and its primary response factor, the Broad-Complex (Br-C)(34,44). In this regulatory cascade, ecdysone functions as a potent transcriptional repressor of miR-34, and treatment with 20-hydroxyecdysone (20-HE) leads to a significant decrease in both mature miR-34 and its primary transcript. To determine how miR-34 expression is modulated by psychosocial stress, we examined the levels of Broad-Complex (Br-C), a hormone-responsive transcription factor previously shown to repress miR-34 transcription during developmental transitions and stress-associated signaling(44). In our study, Br-C expression was significantly elevated by 7.557 ± 2.615-fold in the head tissue of stressed male flies and by 2.320 ± 0.2-fold in females, indicating that miR-34 is transcriptionally repressed under stress, at least in part through Br-C–mediated regulatory mechanisms (Fig. 3a-b). Consistent with this, mature miR-34 levels were reduced following stress exposure, indicating coordinated downregulation at the level of transcription and processing. We also wanted to explore how ecdysone regulates Broad and miR-34 in cell culture, so Kc167 cells were treated with ecdysone (20E) for 48 hours, after which the cells were harvested and RNA was extracted. In corroboration of our findings in *Drosophila*, the levels of both Broad Complex and the primary miR-34 transcript were downregulated in Kc167 cells at 24 and 48 hours of ecdysone treatment, indicating that this regulatory axis may mediate miR-34-associated stress responses (Fig. 3d-e). To further validate the role of Broad complex in the regulation of stress-responsive depression-like behavior, we knocked down Broad in 5-HT1B neurons in the fly brain and observed their behavioral outcomes upon exposure to a stress regime (Fig. 3f). Functional knockdown of *Broad* led to improved stress tolerance in flies, with a 3.4 times and 5.06 times increase in sucrose preference in males and females, respectively (Fig. 3 g-h), 9.18 times and 14.83 times increase in swimming time in males and females respectively indicating amelioration of despair-like state (Fig. 3 i-j) and, 6.96 times and 14.74 times increase in total distance covered in open field by male and female flies respectively, exhibiting lowered anxiety-like phenotype (Fig. 3 k-l). Taken together, this implies that miR-34 and its transcriptional repressor Broad tightly regulate responses to a combination of physical and social stressors, thereby altering stress vulnerability.

### MiR-34 overexpression confers behavioral resilience to stress

**Fig. 4.**
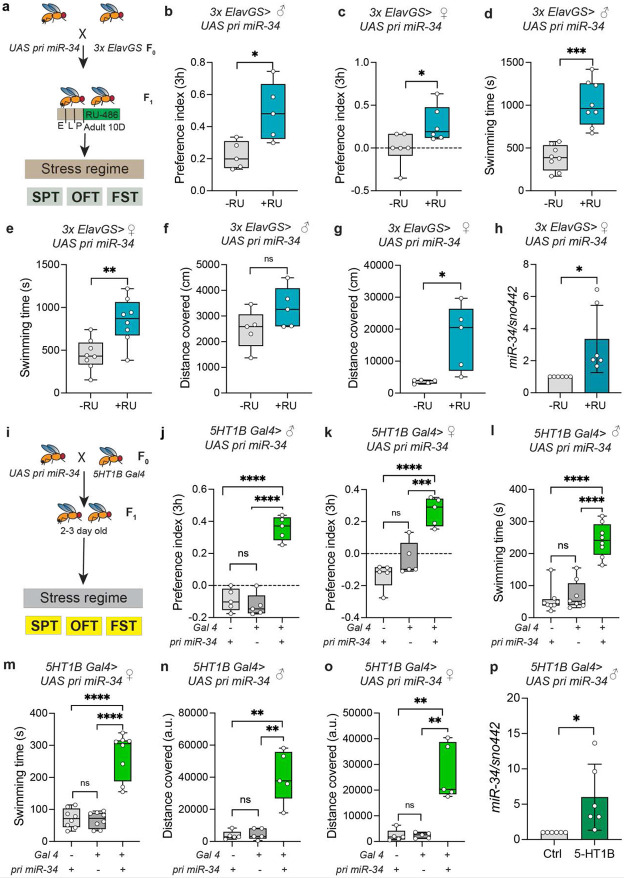
Pan neuronal overexpression of miR-34 confers partial resilience against stress, whereas overexpression in 5-HT1B neurons confers complete alleviation of the stress response. **(a)** A diagram representing the scheme employed for the overexpression of miR-34 using the 3xElavGS. *3xElavGS>UAS pri-miR-34* flies were fed RU-486 during adulthood for 10 days, while controls were fed on a vehicle containing food, following which they were exposed to the stress regime. Their behavior was then assessed through SPT, OFT, and FST. **(b-c)** PI increases in +RU flies, indicating relief from anhedonia upon overexpression of miR-34 in a pan-neuronal manner. Each data point indicates the PI of a group of 10 flies. **(d-e)** Latency to absolute immobility of *3xElavGS>UAS pri-miR-34* male **(d)** and female **(e)** flies significantly increased upon feeding food containing RU-486, indicating resilience from behavioral despair (n=8). **(f-g)** Exploratory behavior of female **(g)** *3xElavGS>UAS pri-miR-34* flies fed on RU-containing food increased in comparison to their controls, while no significant difference was observed in males **(f)** (n=5). The box plot shows the means of each group, with the box spanning from the first to the third quartile and whiskers extending from the minimum to the maximum data points. **(h)** RNA was extracted from the heads of flies fed on RU-486 or solvent-containing food, which was then used for miR-34 quantification using RT-PCR. **(i)** Schematic of the genetic cross and experimental workflow. *UAS-miR-34* males were crossed with *5HT1B-Gal4* females to generate F1 progeny (2–3 days old), which were then subjected to a standardized stress paradigm followed by behavioral assays: Sucrose Preference Test (SPT), Forced Swim Test (FST), and Open Field Test (OFT). **(j-k)** Sucrose preference indices in stressed male **(j)** and female **(k)** of *5-HT1B>UAS pri-miR-34* flies. Overexpression of miR-34 in 5HT1B neurons significantly increased sucrose preference in both sexes compared to controls. **(l–m)** Forced swim test performance showing swimming time in stressed male **(l)** and female **(m)** flies, indicating helplessness-like behavior. miR-34 overexpression rescued stress-induced reductions in latency to absolute immobility in both sexes. **(n–o)** Open field test distance traveled by stressed male **(n)** and female **(o)** flies. miR-34 overexpression significantly increased locomotor activity, indicating restored exploratory behavior. **(p)** qRT-PCR analysis showing miR-34 expression in 5HT1B neurons. MiR-34 levels were elevated in *5HT1B-Gal4>UAS pri-miR-34* flies compared to controls. Data are shown as box-and-whisker plots with individual data points overlaid. Statistical comparisons were made using one-way ANOVA with Bonferroni’s correction (panels B–G) or unpaired t-test with Welch’s correction (panel H). *ns*, not significant; *p < 0.05, **p< 0.01, ***p< 0.005, ****p< 0.001 (****). Genotypes of strains used in this figure **(b-h)** *3XElavGS>UAS pri-miR-34: P{elav-Switch.O}GS -1A / +; P{elav-Switch.O}GS-3A, P{elav-Switch.O} GSG301/P{y[+t7.7] w[+mC]=UAS-LUC-mir-34.T}attP2,* **(j-p)** *5-HT1B>UAS pri-miR-34*: *+/+;P{w[+mC]=5-HT1B-GAL4.Y}3/w[1118]; P{y[+t7.7] w[+mC]=UAS-LUC-mir-34.T}attP2*.

To address whether increasing the dosage of miR-34 in the adult brain is sufficient to promote stress resilience, we used the inducible *3×ElavGS* driver to pan-neuronally overexpress miR-34 in adult flies. Flies carrying *UAS-miR-34* under control of *3×ElavGS* were reared on standard food and, beginning at eclosion, and placed on food containing RU-486 (or solvent) for 10 days to induce adult-specific neuronal miR-34 expression (Fig. 4a). Following this induction period, flies were subjected to a standardized stress regime, and behavior was assessed using three assays: the sucrose preference test (SPT), forced swimming test (FST), and open field test (OFT). In the SPT, RU-induced (miR-34 overexpressing) male flies showed an increase in PI from 0.2205 ± 0.08639 of non-induced stressed controls to 0.4918 ± 0.1802 for flies on +RU food (Fig. 4b). Similarly, in females, miR-34 overexpression led to a significant increase in sucrose preference under stress compared to vehicle controls (−RU=−0.004743 ± 0.1882, +RU=0.2781 ± 0.2085) (Fig. 4c), indicating preservation of reward-seeking behavior. In the forced swimming test, miR-34 overexpressing males exhibited markedly longer swimming times post-stress than non-induced controls (−RU = 386.6 ± 147.5, +RU = 1019 ± 264.6) (Fig. 4d), consistent with enhanced coping with stress. Females overexpressing miR-34 also swam significantly more than their non-induced counterparts as swimming time increased from 448.9 ± 179.8 to 847.0 ± 262.5 (Fig. 4e), suggesting that miR-34 overexpression strengthens stress-coping behavior across sexes. In the OFT, we quantified exploration by measuring total distance traveled. While control (−RU) and miR-34 overexpression (+RU) males did not differ significantly in their stress-induced decline in movement (ns) (Fig. 4f), overexpressing females traversed a significantly greater distance after stress compared to non-induced stressed females (−RU = 3590 ± 571.5, +RU=17442 ± 10195) (Fig. 4g), indicating protection from stress-induced hypoactivity or anxiety-like behavior. To validate the overexpression of miR-34, we measured mature miR-34 levels in the head tissue of female flies and found a robust upregulation of 3.353 ± 2.095-fold in the +RU group relative to −RU (Fig. 4h).

Overexpressing miR-34 in 5-HT1B-expressing neurons via 5-HT1B-GAL4 drive resulted in a complete rescue of stress-induced behavioral deficits across all three assays (Fig. 4i). Stressed *5-HT1B > miR-34* flies showed sucrose preference indices indistinguishable from non-stressed controls, in direct contrast to stressed driver- or UAS-only controls (*5-HT1B>UAS miR-34* males have PI of 0.3573 ± 0.07469 and females showed PI of 0.2638 ± 0.08802 while *5-HT1B>+/+* males and females showed PI of −0.1230 ± 0.07141 and −0.03439 ± 0.1035 respectively)(Fig. 4j-k). In the forced-swim test, both males and females with miR-34 overexpression maintained high levels of active swimming under stress, matching baseline levels with males swimming for 242.0 ± 52.12 seconds and females swimming for 268.3 ± 71.00, in contrast to baseline swimming times of *5-HT1B>+/+* and *+/+>UAS miR-34* stressed flies (*5-HT1B>+/+*; males: 69.75 ± 44.20s, females: 67.25 ± 24.93s; *+/+>UAS miR-34 males*: 55.25 ± 39.47s, females: 72.13 ± 29.82s) (Fig. 4l-m). Likewise, in the open-field assay, miR-34 overexpressors failed to show the stress-associated decline in exploration seen in controls (males covered 40590 ± 15919 a.u. and females covered 26897 ± 10864 a.u.) (Fig. 4n-o). In contrast, *5-HT1B>+/+* stressed males covered 4640 ± 3398 a.u. and females covered 2373 ± 1105 a.u., and *+/+>UAS miR-34* stressed males covered 3816 ± 2689 a.u and females covered 2344 ± 2313 a.u distance. qPCR confirmed a robust induction of mature miR-34 in heads of the *5-HT1B>miR-34* genotype following RU administration, validating the efficacy of overexpression in the targeted neurons (Fig. 4p). *Drosophila* 5-HT1B receptors in mushroom-body γ-lobe Kenyon cells have been shown to control behavioral inactivity in a depression-like state induced by vibration stress, and 5-HT1B knockdown precluded the stress-induced inactivity phenotype. More broadly, enhanced serotonin signaling via 5-HT1B neurons has been shown to reverse suppressive behavioral states: for instance, activation of 5-HT1B neurons in the ellipsoid body promotes recovery of aggression and motivation in “loser” flies (45). By contrast, overexpression in Tyrosine hydroxylase-positive serotonergic neurons also conferred resilience, but to a lesser degree. miR-34 overexpressing males and females displayed significantly elevated sucrose preference under stress compared to controls. Sucrose preference in *Trh Gal4>UAS miR-34* was found to be 0.02634±0.04849 in males and 0.05678 ±0.1372 in females, while preference indices for their genetically stressed controls were found to be −0.08947 ± 0.06971 in males and −0.1360 ± 0.07975 in females (Fig. S1b-c). In swimming behavior, only females showed a marked increase in active swimming time, from 90.20 ± 20.52 to 301.6 ± 107.0 seconds (Fig. S1d and S1e), whereas males did not differ significantly from controls. In the open-field test, both sexes of miR-34 overexpressing flies traversed significantly more distance under stress than their non-overexpressing counterparts (*Trh Gal4>UAS miR-34* males 11027±2501, females 17045 ± 4202 while their respective controls traveled 4640 ± 3398 a.u. and 2373 ± 1105 a.u.) (Fig. S1f-g), indicating enhanced locomotor persistence or exploratory drive. When miR-34 was overexpressed in the mushroom body, including the alpha, beta, and gamma lobes, using the inducible Mushroom body GAL4 driver for 10 days, the effects on stress resilience were more subtle and sex-dependent. In sucrose preference, RU-induced females but not males demonstrated a modest but significant increase in preference under stress from −0.3427 ± 0.3369 in uninduced (−RU) conditions to 0.2004 ± 0.2036 in induced (+RU) conditions in preference indices under stress (Fig. S1i-j). In the swim test, only females fed on the +RU diet exhibited significantly longer swimming times after stress, which increased from 154.0 ± 66.00 in uninduced (−RU) conditions to 388.6 ± 112.5 in induced (+RU) conditions after stress exposure in females (p < 0.01) (Fig. S1k-l). In the open-field assay, +RU males traveled a greater distance under stress relative to controls (distance traveled increased from 13120 ± 1983 a.u. in −RU males to 31022 ± 10803 a.u. in +RU males) (Fig. S1m-n), while females did not differ significantly. The mushroom body (MB) in *Drosophila* is a central integrative hub composed of intrinsic Kenyon cells, long known for its role in associative learning and memory(46). Beyond learning, MB circuits also mediate internal state and motivational behavior: for example, serotonergic signaling via 5-HT1A and 5-HT1B receptors across different MB lobes has been implicated in modulating the “depression-like” state induced by chronic stress(38). Neurons that express tryptophan hydroxylase, the rate-limiting enzyme in serotonin synthesis, are another key component of serotonergic circuitry in the fly brain(47). These neurons produce and release serotonin, thereby modulating a wide range of behaviors. For instance, subsets of tyrosine hydroxylase neurons have been shown to regulate development and endocrine transitions by innervating the prothoracic gland, linking nutrient status to steroid hormone biosynthesis(48). In adult behavior, serotonergic neurons contribute more broadly to locomotion, social motivation, and neuronal homeostasis (49). Together, these data strongly implicate that neuronal overexpression of miR-34 provides behavioral resilience, as flies with miR-34 overexpressed throughout their neurons maintain greater hedonic drive, more active coping, and preserved exploratory behaviors under stress. 5-HT1B neurons act as a primary locus for miR-34–mediated stress protection. The magnitude and completeness of the behavioral rescue in these cells far exceed that seen in tyrosine hydroxylase neurons or mushroom-body neurons. This suggests that miR-34’s ability to mitigate stress-induced anhedonia, behavioral inactivity, and reduced exploration may largely operate through 5-HT1B-expressing serotonergic circuits.

### MiR-34 knockout flies show increased susceptibility to stress

**Fig. 5.**
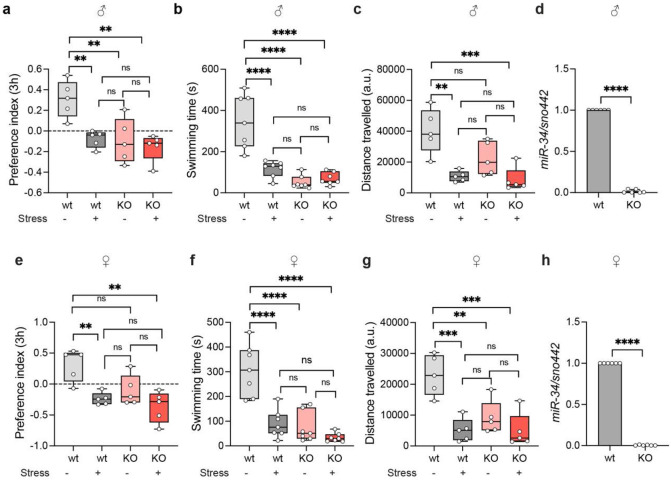
miR-34 knockout abolishes resilience to stress-induced behavioral deficits in both sexes. **(a–c)** Sucrose preference indices in unstressed and stressed wild-type (*wt*) and miR-34 knockout (KO) males **(a)**, swimming time in forced swim test in *wt* and *miR-34*^*KO*^ males **(b)**, and exploratory activity in open field arena in wt and *miR-34*^*KO*^ males **(c)**. **(d)** qRT-PCR showing miR-34 depletion in KO flies, normalized to wild type controls. **(e-g)** Sucrose preference indices in *wt* and *miR-34*^*KO*^ female flies in the presence and absence of psychosocial stressors **(e)**, swimming performance in the forced swim test by *wt* and *miR-34*^*KO*^ female flies **(f)**, and exploratory activity of *wt* and *miR-34*^*KO*^ female flies before and after stress **(g)**. While stress reduced sucrose preference in *wt* flies of both sexes, *miR-34*^*KO*^ flies showed low preference irrespective of stress, with no further stress-induced reduction. Stress significantly decreased swimming time in *wt* flies, whereas *miR-34*^*KO*^ flies exhibited reduced swimming times even without stress and showed no additional decline upon stress. Exposure to a stress regime reduced exploratory activity in *wt* flies of both sexes, whereas *miR-34*^*KO*^ flies displayed consistently low exploration regardless of stress exposure. Data are presented as box-and-whisker plots with individual data points overlaid. Statistical comparisons were performed using one-way ANOVA with Bonferroni’s correction **(a-c, e-g)** and T-test with Welch’s correction **(d and h)**. ns, not significant; *p < 0.05, **p < 0.01, ***p < 0.005, ****p < 0.001. Genotypes used in figure **(a-c)** and **(e-g)** panels 1 and 2 5905 (*w*^*1118*^*)*, panels 3 and 4 *mir-34*^−/−^, *miR-34* ^−/−^ in BDSC 5905 homogenous genetic background.

Loss of miR-34 (miR-34 knockout, *miR-34^KO^*) produces a striking “stress-like” behavioral phenotype, even in the absence of any externally imposed stressor. 2–3-day-old male and female *miR-34^KO^* flies exhibit significantly reduced sucrose preference (females −0.1056 ± 0.2465, males −0.09647 ± 0.2163) compared to wild-type (WT) controls (PI 0.3136 ± 0.2599 in females and 0.3097 ± 0.1786 in males) (Fig. 5a, e) without exposure to a stress regime, indicating a baseline deficit in hedonic or reward-seeking behavior. Upon exposure to stress regime, preference indices of both *wt* and *miR-34^KO^* group showed pronounced anhedonia (wt −0.2389 ± 0.1004 and −0.07802 ± 0.08212, females and males respectively, and *miR-34^KO^* −0.3664 ± 0.2520 and −0.1572 ± 0.1341, females and males respectively) (Fig. 5a, e).In the forced-swim test, *miR-34^KO^* flies show markedly lower active swimming time than *wt* (control unstressed females swim for 307.0 ± 104.3s and males swim for 346.9 ± 130.2s, while *miR-34^KO^* unstressed females swim for 89.71 ± 56.49s and males swim for 116.1±38.73), mirroring the enhanced helplessness behavior typically observed in stressed wild type flies (Fig. 5b, f). In the open-field assay, both male and female *miR-34^KO^* flies traveled significantly less distance than *wild type* in unstressed conditions. Wild-type females traveled 22977 ± 6559 a.u., whereas *miR-34^KO^* female flies traveled 9151 ± 5433 a.u., and *wild-type* males traveled 40072 ± 14437 a.u., and *miR-34^KO^* male flies traveled 22425 ± 10867 a.u. (Fig. 5c, g), indicating reduced exploratory locomotion or spontaneous activity. Importantly, when exposed to stress, the behavior of *miR-34^KO^* flies does not worsen significantly relative to their unstressed *miR-34^KO^* baseline, suggesting they are already stressed in the absence of stressors. To confirm miR-34 knockout in the head tissue of flies, we performed a qRT-PCR to quantify *miR-34* levels in wild-type and knockout flies and found that knockout flies show a complete absence of miR-34 in head extracts and a 63.5-fold decrease of miR-34 in males and ~202-fold decrease in females (Fig. 5d, h). This supports our hypothesis that miR-34 is required to maintain behavioral homeostasis, and that its absence alone is sufficient to trigger a phenotype that phenocopies chronic stress in wild-type animals.

### *CG7611/Mel* is a bona fide target of miR-34 and critically regulates depression-like behavior in response to stress

*CG7611* is a fly ortholog of mammalian *WDR26* (WD repeat domain 26) and is predicted to be involved in G protein signaling pathways. In mammals, WDR26 scaffolds Gβγ→PLCβ signaling by facilitating PLCβ recruitment to the membrane, thereby influencing MAPK signaling(50-52). It is also predicted to be part of the CTLH complex, an E3 ubiquitin ligase complex that modulates proteostasis(53). A recent study has proposed that CG7611 functions as a candidate adapter for the CTLH complex in a context-dependent manner (54). As we found that miR-34 abundance influences stress resilience, we sought to identify novel miR-34 targets that could regulate stress responses. Overexpression of miR-34 in 5-HT1B neurons significantly alleviates depression-like behavior; thus, we hypothesized that knockdown of the functionally relevant target(s) would phenocopy the behavior of miR-34-overexpressing flies. To identify the crucial targets underlying regulation of stress-responsive behavior, we knocked down a set of miR-34 targets, *Su(z)*, *Pcl, Gawky*, *neurexin IV*, and *CG7611* in the 5-HT1B neurons and assessed their behavioral outcomes upon exposure to stress.

**Fig.6.**
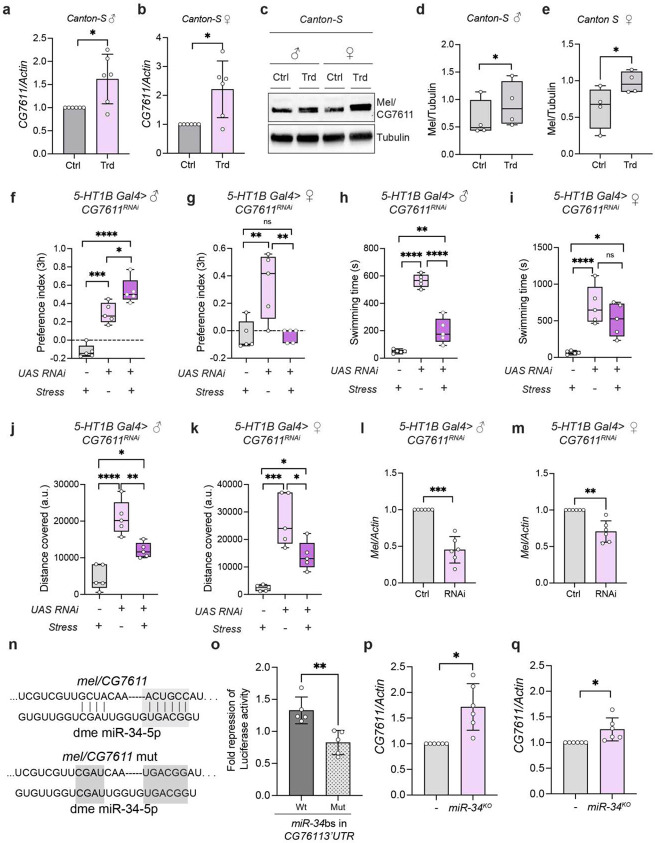
*Mel* is upregulated by stress, and its knockdown in 5-HT1B neurons modulates stress-induced behavioral responses in a sex-dependent manner. **(a–b)** Quantitative real time RT–PCR analysis of *CG7611* expression in stressed (Trd) and control (Ctrl) *Canton-S* males **(a)** and females **(b)**, showing upregulation of *CG7611* in both sexes. **(c)** Representative immunoblots showing CG7611/Mel protein levels in Trd and Ctrl *Canton-S* males and females, with Tubulin as loading control. **(d–e)** Quantification of CG7611/Mel protein levels normalized to Tubulin in males **(d)** and females **(e)**, confirming increased expression upon stress. **(f–g)** Sucrose PI (3h) in stressed male **(f)** and female **(g)** *5-HT1B Gal4>UAS CG7611*^*RNAi*^ flies. *CG7611/mel* knockdown significantly increased sucrose preference in stressed males, while females showed no significant change. **(h–i)** FST performance in males **(h)** and females **(i)**, reflecting helplessness-like behavior. *CG7611/mel*^*RNAi*^ increased swimming time in stressed males and females. **(j–k)** OFT showing total distance traveled in stressed males **(j)** and females **(k)**. *CG7611/mel* knockdown enhanced exploratory activity in stressed males and females. **(l–m)** qRT–PCR validation of *CG7611/mel* knockdown in male **(l)** and female **(m)** *5-HT1B Gal4>UAS CG7611*^*RNAi*^ flies using total RNA isolated from head samples. **(n)** Schematic of the wild type (*wt*) and mutant (*mut*) miR-34 binding site (miR-34bs) in the 3'UTR of *CG7611/mel*. **(o)** Luciferase reporter assay showing reduced activity of wild-type compared to mutant *CG7611* miR-34 binding site constructs. **(p–q)** Quantitative real time RT–PCR analysis of *CG7611/mel* expression in *miR-34*^*KO*^ males **(p)** and females **(q)**, showing increased *CG7611/mel* expression upon miR-34 loss. Data are presented as box-and-whisker plots with individual data points overlaid. Statistical comparisons were performed using unpaired t-tests with Welch’s correction (a–b, d–e, l–m, o–q) or one-way ANOVA with Bonferroni’s correction (F–K). ns, not significant; p < 0.05 (*), p < 0.01 (**)*, p < 0.005* (***), p < 0.001 (****). Genotypes used in this figure **(a-b)** *Canton S*, **(f-m)** +/y[1] sc[*] v[1] sev[21]; P{y[+t7.7] v[+t1.8]=TRiP.HMS05669}attP40/CyO; P{w[+mC]=5-HT1B-GAL4.Y}3/+, **(p-q)** *mir-34*^−/−^, *miR-34*^−/−^ in 5905 homogenous genetic background in panel 2, 5905 (wt) in panel 1.

*CG7611* is predicted to be a target of miR-34 by bioinformatic screening using TargetScan. In vivo, we observed that *CG7611* mRNA and protein levels were upregulated in fly brains following stress, consistent with stress-induced suppression of miR-34 (Fig. 6a-e). *CG7611* mRNA is increased by 61.8% in males and 121.1% in female flies upon exposure to the stress regime (Fig. 6a-b), while protein levels show a 42.55% increase in males and females exhibit a 54.08% increase in stressed conditions. (Fig. 6c-e). Importantly, knockdown of *CG7611* in the 5-HT1B neurons conferred behavioral resilience, such that *CG7611* knockdown male flies exposed to the same stress regime showed significantly improved sucrose preference, recapitulative of unstressed conditions (preference indices in *5HT1B>CG7611^RNAi^* males is 0.5383 ± 0.1378 after being subjected to stress regime, stressed genetic controls showed PI of −0.1230±0.07141) however, female flies did not exhibit relief from stress-associated anhedonia upon *CG6511* knockdown (Fig. 6f-g). The lack of relief could be due to insufficient knockdown or the involvement of female-specific mRNA targets of miR-34. In FST *CG7611* knockdown in both males and females showed increased active swimming (swimming time increase to 197.8 ± 91.75s in males whereas stressed genetic controls swam for 49.00 ± 13.42, in females swimming time was 513.2 ± 225.9s whereas stressed genetic controls swam for 65.40 ± 24.09s), and more sustained exploratory activity compared to stressed genetic control flies (*5HT1B Gal4>CG7611^RNAi^* males: 12035 ± 2051, females: 14060 ± 5194, *5HT1B Gal4>+/+* males: 4640 ± 3398, females: 2373 ± 1105 pixels) (Fig. 6f-k). Knockdown of *CG7611* was confirmed by quantitative real-time RT-PCR, showing a 54.74 ± 7% reduction in *CG7611* mRNA levels in males and only a 29.53 ± 5.9% reduction in females (Fig. 6l, m). These results strongly suggest that *CG7611* acts downstream of miR-34 to mediate stress susceptibility, and that repression of *CG7611* is functionally beneficial for stress resilience; its derepression (as seen under stress) contributes to behavioral vulnerability. Since this study has uncovered a new role for the previously computationally predicted *CG7611*, we have named this gene *melancholy (mel)*, as its dosage is associated with a behavioral state characterized by sadness.

We further confirmed that *CG7611/mel* is a bona fide target of miR-34 in Kc167 cells using a luciferase dual reporter assay. The 3’UTR of *Mel*/*CG7611* is predicted to have a 5-mer binding site for miR-34 (Fig. 6n). Kc167 cells were transfected with a luciferase reporter plasmid containing wild-type and mutant miR-34 binding sites present on the endogenous 3’UTR of *CG7611,* along with a UAS plasmid expressing *Drosophila pri-miR-34* in the presence of Tubulin Gal4. Luciferase assay results revealed that miR-34 binds to the wild-type *CG7611* 3’UTR fragment, resulting in repression of luciferase reporter activity, which is ameliorated when the wild-type site is mutated, leading to loss of miR-34 binding (Fig. 6o). Additionally, *CG7611/mel* mRNA levels are upregulated in *miR-34^KO^* flies by 0.718-fold in male flies and 0.265-fold increase in female flies, indicating *CG7611/mel* is regulated in vivo by miR-34 (Fig. 6p-q).

We also examined the effects of other predicted miR-34 targets. Knockdown of *Su(Z)* increased stress resilience specifically in males, as observed by higher sucrose preference (PI in males increased by 617.6% ± 381.5% in comparison to stressed controls, in females PI decreased by 251.6% ± 890% compared to controls, ns), increased swimming time in the FST(*Su (Z)^RNAi^* males: 821.1% ± 702.1% increase compared to controls, females 23.3% ± 108.1% in comparison to controls), and greater exploratory activity (males show an increase of 355.5% ± 318.8% swimming time in comparison to controls while females show a 58.9% ± 143.7% increase compared to controls) (Fig. S2a-g). Conversely, knockdown of *Polycomb-like* (*Pcl*) did not impact stress resilience in both sexes (sucrose preference *5HT1B Gal4>Pcl^RNAi^* males show a 77.8% ± 177.4% increase in comparison to stressed controls while females exhibit a 298.1% ± 1163.6% decrease in comparison to stressed control flies)(Fig. S2i-j). Only females showed an increase in swimming time (215.6% ± 216.5% increase in males relative to controls, while in females, swimming time increased by 2121.7% ± 1429.7% compared to their control flies) (Fig. S2k-l). No relief was observed in case of exploratory activity deficits (in males, exploratory distance was decreased 31.6% ± 78.3%, and in females, increased by 23.2% ± 70.0% compared to controls) (Fig. S2m-n).

### Overexpression of *melancholy (mel)* in 5-HT1B neurons heightens stress responses and drives coordinated extracellular proteomic remodeling

**Figure 7.**
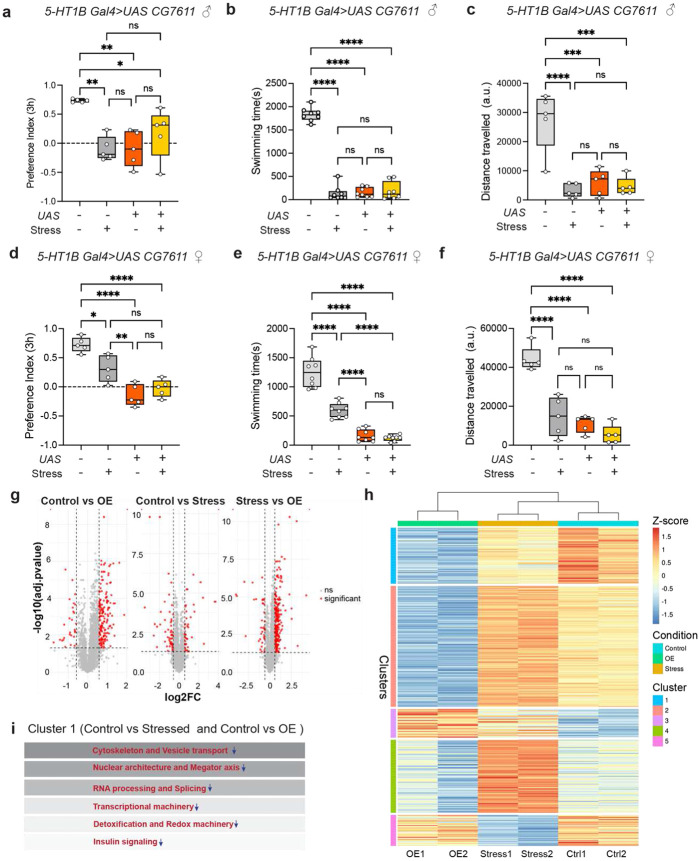
Overexpression of *Mel* in 5-HT1B neurons regulates stress-induced behavioral responses. Males and females carrying *5-HT1B-Gal4 > UAS-Flag Mel* transgene were analyzed under non-stress (−) or stress (+) conditions, with or without *Mel* overexpression as indicated. PI over 3 h, total swimming time, and total distance traveled were quantified. **(a-c** In males, stress significantly reduced PI, swimming time, and distance traveled compared to non-stressed controls, and *Mel* overexpression phenocopied these effects even in the absence of stress. **(d–f)** In females, stress similarly impaired behavioral performance, and *Mel* overexpression significantly exacerbated these responses, even under non-stress conditions. Data are presented as box-and-whisker plots showing median, interquartile range, and range, with individual data points overlaid. Statistical significance was determined using multiple comparisons tests as indicated; ns, not significant; *P < 0.05, **P < 0.01, ***P < 0.001, ****P < 0.0001. **(g)** Volcano plots showing log_2_ fold change versus −log_10_ (adjusted p value) for the three pairwise comparisons (Stress vs OE, Control vs Stress, and Control vs OE). Proteins with adjusted p value < 0.05 and ∣log_2_FC∣ > 0.58 are highlighted as significant (red), while non-significant proteins are shown in grey; dashed lines indicate the significance and fold change thresholds. **(h)** Heatmap of Z–score–scaled protein intensities for significant proteins across the six samples (OE1, OE2, Stress1, Stress2, Control1, Control2). Proteins were partitioned into up to five K-means clusters based on their expression profiles, and rows are ordered by cluster membership, with column annotations indicating sample condition and row annotations indicating cluster assignment. (I) Schematic representing the modules of proteins identified in Cluster 1. Genotypes of strains used in this figure **(a-i)** *5HT1B Gal4> UAS CG7611*: w[*]; UAS Flag v}attP40/CyO; P{w[+mC]=5-HT1B-GAL4.Y}3/+.

To examine whether overexpression of *mel* mimics the behavioral phenotype associated with miR-34 depletion, we generated a *UAS-mel* line that expresses a flag-tagged *Mel* cDNA. Balanced flies were crossed to *5-HT1B GAL4* or *3xElavGS (3X ElavGS)* to specifically overexpress *mel* in *5-HT1B* neurons and throughout all neurons of the brain, respectively. To determine if increased dosage of *mel* is sufficient to drive affective dysfunction, we assessed behavioral phenotypes in the absence of external environmental stress. Remarkably, in both males and females, the targeted overexpression of *Mel* under basal conditions was sufficient to recapitulate the behavioral hallmarks of chronic stress (Fig. 7a-f). In the SPT, non-stressed flies overexpressing *mel* exhibited a significant reduction in PI (Males, −0.09379±0.3062; Females, 0.1477±0.1853 ) compared to non-stressed genetic controls (Males, 0.7319 ± 0.02803; Females, 0.7240 ± 0.1274), reaching levels comparable to those of flies subjected to the stress paradigm having PI of −0.09521 ± 0.2077 in males and 0.3121 ± 0.2289 in females (Fig. 7 a, d). Similarly, in the FST, *mel* overexpression alone induced a profound state of behavioral despair, with swimming times drastically reduced to levels indistinguishable from those of stressed control groups (*5-HT1B> UAS mel*: Males, 154.1 ± 103.7; Females, 163.1 ± 101.3; stressed 5-HT1B> +/+: Males, 138.6±158.4; Females, 596.9 ± 126.6) (Fig. 7b, e). This genetic "mimicry" of the stress state extended to exploratory activity, where *CG7611* gain-of-function significantly diminished the total distance travelled in the Open Field Test (OFT) in both sexes (*5-HT1B> UAS mel*: Males, 5932 ± 4403 a.u.; Females, 10878 ± 4343 a.u; stressed 5-HT1B> +/+: Males, 3257 ± 2357 a.u.; Females, 14641 ± 9973 a.u.) (Fig. 7c, f). Notably, the lack of a significant difference between non-stressed overexpression groups and their stressed counterparts (Fig. 7a-f) suggests that elevated *mel* levels in 5-HT1B neurons may act downstream of or converge upon the same physiological pathways activated by chronic environmental stress. These data identify *mel* as a potent molecular mediator that can independently drive anhedonia-like behavior, despair, and reduced mobility, effectively bypassing the need for external stress.

Upregulation of *mel* likely alters its scaffolding or regulatory functions via its conserved WD domains, possibly disrupting neuronal homeostasis and triggering a stress response in the absence of stressors. Stressed flies behave similarly to non-stressed flies overexpressing *mel*, with no statistically significant difference in their behavioral outcomes upon stress exposure, reinforcing the mechanistic role of *mel* in maintaining optimal stress responses. We also overexpressed *mel* throughout all neurons in adulthood using the *3xElavGS*, and these flies showed increased susceptibility to stress in the absence of stressors, indicating that *mel* overexpression is sufficient to induce stress responses globally in the fly brain (Fig. S3a-h). Sucrose PI in *3x Elav GS>UAS mel* flies decreased upon dietary intake of RU-486 to −0.04461 ± 0.3418 in unstressed males in comparison to unstressed −RU males having PI of 0.4755 ± 0.1483, upon stress −RU group’s PI reduced to −0.3363 ± 0.4676, while +RU group’s preference was found to be −0.2933 ± 0.3871; in females PI of +RU group was −0.3117 ± 0.3907 in the absence of stress and −0.09392 ± 0.3313 after exposure to stress, while −RU group had a PI of 0.3746 ± 0.1817 which reduced to −0.2814 ± 0.2553 upon stress (Fig S3a-b). *Mel* overexpression in all adult neurons also impacted exploratory behavior, with unstressed +RU males covering 18808 ± 2757 distance in arbitrary units (a.u.), comparable to stressed −RU males covering 9632 ± 5675 a.u., while unstressed −RU males cover 26816 ± 4490 a.u. and after stress exposure +RU males covered 14851 ± 6222 a.u.; unstressed +RU females exhibit 5540 ± 2695 a.u, similar to stressed −RU group with 7013 ± 3269 a.u while unstressed −RU females cover 13485 ± 2050 a.u and stressed +RU females cover 4062 ± 2767 a.u. (Fig S3c-d). Similarly, +RU flies showed a decrease in swimming time in FST indicative of a despair-like state (swimming time in unstressed −RU males was 575.0 ± 151.9s which reduced to 281.5 ± 173.1s upon administration of RU-486; swimming time in −RU males reduced to 214.4 ± 160.9s upon exposure to stress regime while +RU males exhibited a further reduction in swimming time to 178.6 ± 116.1s; in females unstressed −RU flies exhibited 419.1 ± 143.8s of swimming time which reduced to 166.8 ± 111.1s upon stress, comparable to +RU unstressed females having swimming time of 186.4 ± 152.5s in unstressed condition and 166.6 ± 121.7s in stressed condition (Fig S3e-f). However, the effect observed upon adult-specific, pan-neuronal overexpression of *mel* was less than the detrimental effect caused by overexpression of *Mel* in 5-HT1B neurons throughout development and into adulthood. Nevertheless, these data indicate that 5-HT-1B cells are critical mediators of stress responses in flies.

To determine whether *Mel* overexpression (OE) recapitulates stress-associated molecular changes, we performed quantitative proteomic profiling of control and stressed *5HT1BGal4>w^1118^* female flies and *5HT1BGal4>UAS mel* flies. We identified 46,151 and 46,044 unique peptides in control *5HT1BGal4>w^1118^* flies, mapping to 5,726 and 5,727 proteins, respectively, of which 39,967 and 39,920 peptides uniquely mapped to single proteins. In stressed *5HT1BGal4>w^1118^* flies, 46,818 and 46,775 peptides were identified, mapping to 5,738 and 5,748 proteins, with 40,569 and 40,518 peptides uniquely mapping to single proteins (Table S4-S6). In contrast, *5HT1BGal4>UAS mel* flies showed 45,240 and 44,524 unique peptides, mapping to 5,712 and 5,709 proteins, of which 39,291 and 38,651 peptides uniquely mapped to single proteins. The volcano plot shows that 168 and 307 proteins were significantly downregulated in *mel*-overexpressing fly heads relative to control and stress conditions, respectively, while only 40 and 43 proteins were upregulated (Fig. 7g). In contrast, the Control vs Stress comparison showed only 45 proteins upregulated in Stress and 75 downregulated as compared to control (Fig. 7g-h, S4a). K-means clustering of protein abundance profiles revealed five functional clusters (Fig. 7h and Table S6). The global proteomic profiles indicate that the control and stressed conditions were more similar than the *mel* overexpressor profiles. This clustering further identified distinct co-regulated protein modules, among which clusters 1 and 3 showed consistent and concordant regulation in both stress and in response to *Mel* overexpression relative to controls (Fig. S4b-c). To examine the associations among the differentially expressed proteins across the three groups, the online database STRING 12 (Search Tool for Retrieval of Interacting Genes/Proteins) was used to map networks across the five clusters (55). In cluster 1, proteins from *mel*-overexpressing flies grouped more closely with stressed genetic controls than with unstressed controls, indicating a shared global proteomic signature between *mel* overexpression and psychosocial stress (Fig 7i and S4b). In this cluster, the protein abundance in *mel*-overexpressing samples and stressed control samples was lower than in unstressed control samples. Moreover, the reduction was more significant upon mel overexpression (Fig. 7h-i). This cluster was enriched in Cytoskeleton and Vesicle transport proteins/genes (Q9V4C1/ *CG1674-PH*, M9PDB4/ *CLIP-190*, A1ZA47/ *Zasp52*, Q7K549/*mlt*, Q9V9S7/*RhoGAP100F*, Q8INK9/ *SelR*, Q9VF66/ *Rbp*, P18091/ *Actn*, Q24400/ *Mlp84B* and A8JQV7/*pyd*), nuclear architecture and megator axis (Q9VE85/*Nup43* and *A1Z8P9Megator*) chromatin and histone proteins (P84051/*His2A* variants, P02283/*His 2B* variants, P84040/His 4 variants, Q05783/*HmgD*), RNA processing and splicing (Q9V3T8/*SC35*, Q9VHC0/*RnpS1*, Q9VMC8/*Phf5a*, Q7K533/*Gbp2*, Q9W1H5/*DCP1*), transcriptional machinery (O97183/*Polr2C*, Q27272 /*Taf9*), detoxification and redox (Q9V674/*Cyp6g1*, Q9V4T5/*Cyp4e1*, and Q8INK9/*SelR*) and systemic insulin signaling(Q7KUD5/*Ilp5*). This profile reflected a brain-specific adaptive reprogramming state characterized by reduced nuclear dynamics, transcriptional throughput, and neuronal plasticity

Cytoskeletal proteins form an important component of both pre- and post-synaptic terminals, and hence, alterations in cytoskeletal organization can lead to structural changes such as the growth or disappearance of pre-existing synapses or the appearance of new synapses, which may ultimately influence changes in neuronal circuits that affect behavior in response to stress (56). Based on KEGG pathway analysis, cluster 1 proteins were involved in nucleocytoplasmic transport (Q9VE85/*Nup43*, A1Z8P9/*Megator*, Q9VHC0/*RnpS1*, Q9VJ12/*Acn* and Q9V3T8/*SC35*). Nup43 and Megator play important roles in stress-induced responses by regulating nuclear architecture and gene expression through interactions with the chromatin of different genes(57). The Acn and RnpS1 proteins are present in an ASAP (Apoptosis- and splicing-associated protein) complex in the nucleus, and the *Drosophila* Acn protein is involved in autophagosome maturation and endosomal trafficking (58). SC35 is a serine/arginine-rich (SR) splicing factor that has been implicated in transcriptional and splicing during cellular stress through its interaction with the long noncoding RNA hsrω. Although SC35 overexpression has been associated with stress, its knockdown is linked to thermal nociception defects(59). In addition, a significant decrease was observed in the protein (P91621) encoded by *still life* (*sif*) in the *mel* overexpressing fly head tissue. P91621 is localized to presynaptic terminals in the central nervous system and neuromuscular junctions and regulates synaptic growth at neuromuscular junctions. Other nucleosome components significantly downregulated upon mel overexpression include Q8MSW9, P84051, and P02283, U3-55K, and Histone core components (H2A and H2B). Since the mammalian ortholog of *mel* is an essential component of the CTLH E3 ligase complex, which is involved in ubiquitin-mediated protein degradation and regulates chromatin accessibility, it is likely that the decrease in nucleosome proteins results from a conserved role of Mel in ubiquitin-mediated degradation. Since the cytoskeleton governs axonal transport, synapse structure, and plasticity, the downregulation of Cluster 1 proteins led to decreased synaptic remodeling and vesicle trafficking.

Taken together, these changes would allow stabilization of existing neural circuits, reduce energy-intensive synaptic turnover, and protect against synaptic degeneration. The downregulation of chromatin-associated and transcriptional machinery reduced the metabolic burden of transcription and potentially stabilized the low-noise gene expression state. Reduced nucleocytoplasmic transport and dampened transcriptional hubs at nuclear pores, potentially leading to reduced activity-dependent transcription and neuronal responsiveness. The data also showed reductions in RNA processing/transport, autophagosome formation, and endosomal trafficking, indicating a global slowdown in intracellular trafficking systems. Since these processes consume a lot of energy, these alterations would mimic a low activity neuronal state that would conserve energy and match protein synthesis and turnover over the shorter term but would impair clearance of damaged proteins/organelles and increase proteotoxic stress with time. We also observed downregulation of insulin signaling, which could reduce growth and metabolic drive in the brain. Overall, these changes reflect a nuclear architecture-mediated control of neuronal state that led to reduced behavioral responsiveness, protection against excitotoxicity, and allowed long-term neuronal survival under stress. These data suggest that *mel* overexpression was sufficient to induce stress and, hence, led to behavioral deficits representative of stressed flies.

Cluster 3 represented the group of proteins significantly upregulated in stress and/or *mel* overexpression compared to unstressed control samples (Fig S4c). These were likely compensatory upregulated mechanisms that increased mitochondrial efficiency, enhanced detoxification, promoted structural stabilization, facilitated selective translation, and maintained nuclear envelope integrity (Fig S4C). These proteins were enriched for biological processes related to mitochondrial electron transport, and the regulation of cytochrome c oxidase (Q9W1N3/*levy*, Q9VIQ8/*COX4*, Q9VMS1/*cype*, Q9VVG5/ *CG7630*, Q9VWD1/*COX6B*, Q9VHS2/*COX7A*, Q8SYJ2/*ND-MLRQ*, P00408/ *mt:CoII*, Q6IHY5/ *COX7AL2*), metabolic flexibility or alternate fuel utilization(A0A0C4DHE7/Gdh, Q9VT15/*CG3088*, Q9VPR3/*CG2794*, Q59E07/*CG2082*), protection against mitochondrial ROS (Q27593/Cyp6a8, Q9VMS1/*cype*, Q9VGT8/*Ugt35C1*, Q9VJ45/*Ugt36E1*), structural stabilization to reinforce a low-plasticity state (P02572/*Act42A*, A1Z8Q0/*Twdlbeta*, Q9VB86/*TwdlT*), select translation module to allow targeted protein synthesis (Q24154/*RpL29*, Q9I7D3/*Capr*) to allow a shift from bulk translation to selective protein synthesis, a nuclear integrity and envelope module (P20240/*Ote*) to stabilize the suppressed nucleus and lastly an extracellular and immune module (Q9VVK5/*Adgf-A*, Q8SXX2/*Ance-3*) to coordinate tissue level adaptation by linking neuronal state to organismal physiology.

The increase in proteins involved in oxidative phosphorylation and metabolic pathways improves the ATP yield per substrate and supports neuronal survival while simultaneously compensating for reduced metabolic flexibility. This adaptation, together with decreased transcription, synaptic activity, and reduced trafficking due to Cluster 1 changes, likely enables efficient, steady energy production to sustain an essential neuronal state. The increased metabolic flexibility module enables amino acid-based metabolism and TCA cycle fueling to compensate for reduced glycolytic demand and reduced insulin signaling. Thus, allowing mitochondria to function under stress conditions and support neuronal survival without a high anabolic load. The upregulation of OXPHOS proteins lead to increased mitochondrial activity and ROS production (60). Hence, to prevent damage from accumulating, levels of select redox and detoxification proteins increased. The structural stabilization proteins (Act42A, Twdlbeta, TwdlT) reinforced existing structures while suppressing dynamic remodeling. These changes likely allowed long-term circuit preservation and stabilized neuronal morphology and extracellular barriers. Since global transcription and RNA processing are reduced, selective translation (Rpl29 and Capr) prioritized survival and stress-response proteins while avoiding the energy waste of global protein synthesis. The nuclear integrity and envelope protein (Ote) stabilized the nuclear envelope and aided its maintenance despite reduced transport and transcription.

Cluster 2 represented the largest group of proteins that were significantly downregulated upon mel overexpression but upregulated in stressed flies compared to the control unstressed group (Fig. S4d). Thus, this cluster would correspond to protein networks altered specifically upon mel overexpression. This cluster was enriched for chromatin regulators (Q9VMJ7/Kdm5, Q9VRP9/Bre1, P08985/His2Av, Q24478/Cp190, Q86BS3/Chro, Q9VGA4/MBD-R2, Q9VP57/pzg, Q9VAA9/JASPer, Q86B87/mod (mdg4)(61,62), transcriptional machinery (P08266 /Polr2B, Q24325/Taf2, Q9V460/Spt5, Q9W420/Spt6, Q94883/Dref, Q9VHM3/M1BP, Q08605/Trl, P05552/Adf1)(63), nuclear pore complex (NPC) components(Q7K2X8/Nup44A, Q9VXE6/Nup153, Q9VKJ3/Nup160, Q8IQV9/Nup205, Q9V463/Nup154, Q9VCW3/Nup133, Q9V466/Nup107, A1YK02/Nup75, Q9XZ06/Nup93-1, A1Z6H7/Gp210) and RNA processing factors (Q9VMR6/Sf2, Q9VSH4/Cpsf6, Q9VKK1/Ge-1, Q9W1F4/thoc5, Q02427/Rbp1), protein translation and ribosome-associated factors (A1ZA22/eif2Bgamma, Q9VRY5/Sbds, Q9VJC7/mEFTs, Q9VFL5 /MetRS-m), signaling, trafficking and vesicle systems(Q9VEA2/Vps39, Q9VKV6/TBC1D16).

This comprises proteins involved in multiple layers of control, including suppression of transcription initiation and elongation, impaired nucleo-cytoplasmic transport, and disruption of NPC-associated transcription hubs, downregulation of RNA maturation and export machinery, reduced translation initiation and ribosome function, and reduced vesicular trafficking and membrane dynamics in the cytoplasm. These data indicate that *mel* drives ubiquitination and degradation of nuclear pore proteins, transcription machinery, and chromatin regulators (61). Taken together, these data indicate that *mel* overexpression induces a proteasome-driven inhibition of nuclear function by targeting chromatin regulators, transcription machinery and nuclear pore components.

Cluster 4 represented proteins significantly upregulated under stress relative to unstressed flies (Fig S4f). These proteins could be classified into different functional modules that collectively reshape stress physiology, and their coordinated upregulation is likely due to a systems-level stress adaptation. The first module comprises of proteins involved in small molecule metabolic processes (P42281/*Acbp2*, Q9VFC8/*GlyS*, A0A0B4KHW3/*Aralar1*, E2QCN9/*GS1-Like*, Q9VLG9/*Argl,* Q9VF53/*Aox1*, Q9VP61/ *AcCoAS*, Q7KSC4/*Mpc1*, Q9V9W4/*CG1774*, Q7KTW9/*AsnS*, Q9VCY8/*AdipR*, E1JJH5/*dlg1*, Q7JUS1/*CG30491*, O97479/*Sodh-1*, Q9W095/*Gk2*, Q7K4Y0/*Desat1*, Q9V4C0/*yellow-h*, Q9VP02/ *CG32444*, Q9I7S8/*Paics*, and Q9VA02/*CG1544*). The increased levels of these proteins shift metabolism towards flexible fuel usage, enable energy storage (glycogen/lipids), and promote ATP production under stress conditions (62-64). These functions provide an adaptive advantage, allowing cells to function despite stress. The second module includes proteins involved in proteostasis and the heat shock response (P02517/*Hsp26*, P02516/*Hsp23*, P02515/*Hsp22*, O97125/*Hsp68*, P02518/*Hsp27*, Q8IN44/*TotA*, and Q8IN43/*TotC).* These proteins act as molecular chaperones, preventing protein misfolding and aggregation. These proteins also promote the refolding or degradation of damaged proteins under oxidative stress and heat shock (65-67). Thus, elevated levels of these proteins help maintain proteome integrity in stressed flies. The third module includes proteins involved in vesicular trafficking and proteostasis coupling (Q9W0H9/*Rabex-5*, Q9VIW6/*Rab9*, Q7JXV9/*Vps25*). These proteins enhance cellular cleanup systems and prevent toxic buildup during stress by regulating endocytosis, recycling, lysosomal degradation, and the removal of damaged proteins and organelles (68-70). The fourth module includes proteins involved in membrane remodeling (Q7K4Y0/*Desat1*, P42281/*Acbp2,* and A1Z8N1/*Tret1-1*). The increased levels of these proteins improve cellular stability and osmoprotection by adjusting membrane fluidity, stabilizing lipid transport and storage, and maintaining carbohydrate transport(71,72). The fifth module includes proteins involved in detoxification and redox systems (Q9VF53/*Aox1*, Q9W1E9/*Fmo-1*, P19967/*Cyt-b5-r*, P82713/*Cyp309a2*, and Q9V6H1/*Cyp9h1*). These proteins reduce the oxidative stress burden and protect lipids, DNA, and proteins under chronic stress by detoxifying reactive oxygen species (ROS) and maintaining redox balance (25,26). Consistent with miR-34 downregulation upon stress, we also observed upregulation of two of its predicted targets, *Dlg1* (Discs large 1) and *Mpc1* (Mitochondrial pyruvate carrier 1), in samples from stressed flies as compared to unstressed flies. Dlg1 is a membrane-associated guanylate kinase (MAGUK) protein that regulates epithelial cell polarity, cell junction structure, and synaptic plasticity at neuromuscular junctions (73,74). Mpc1 is predicted to be involved in the transport of pyruvate across the mitochondrial inner membrane. Upregulation of the Mpc complex helps manage metabolic inflexibility and prevent lactate accumulation (75). Taken together, the proteins in these modules function as a coordinated adaptive network to limit the damage and provide structural resilience and protein quality control.

Cluster 5 represented proteins significantly decreased in stressed samples compared with control and *mel*-overexpressed samples (Fig. S4e). This cluster was enriched in cytoskeletal actin-binding proteins (P14318/*Mp20*, E1JHJ3/*Mhc*, Q9I7J0/*CG5023*, Q07171/*Gel*, Q9VI25/*Neurochondrin*, C0HK95/*fau*), transcriptional and RNA regulatory machinery (Q9W278/*MED16*, P07909/*Hrb98DE*, Q8T8R1/*CNBP*, Q9VHP0/*bel*, P26802/*Dbp73D*), metabolic enzymes and energy flux (P20007/*Pepck*, P54398/*Fbp2*, Q9VG74/*NANS*), Calcium excitability (A0A0B4LGB7/*SERCA*), vesicular trafficking and membrane dynamics (Q9V359/Vps28) and some secreted proteins (P54195/*Obp28a*, P14318/*Mp20*, Q8IQU1/*verm*).

Downregulation of cytoskeletal proteins would reduce cytoskeletal remodeling, limit energy-intensive cellular movements, and conserve energy. Amongst these, Q07171/Gelsolin regulates actin filament dynamics by severing them to maintain structural integrity. Knockdown of this protein leads to increased sensitivity to environmental stress and toxins(76). Downregulation of the transcriptional and RNA-processing machinery would limit transcription of growth- and proliferation-related mRNAs and prevent the accumulation of misprocessed mRNAs under stress conditions. Downregulation of metabolic enzymes would suppress gluconeogenesis and anabolic metabolism, thereby limiting biosynthetic pathways. Downregulation of SERCA would alter Endoplasmic reticulum (ER) calcium uptake and signaling dynamics, leading to reduced ATP consumption and reduced cellular excitability. A reduction in vesicular trafficking would limit membrane turnover and receptor signaling, thereby contributing to cellular slowdown. The overall pattern reflects a stress-adaptation strategy where there is a shift from growth, proliferation, and high metabolic flux towards maintenance, damage control, and energy efficiency.

Mechanistically, our findings implicate *Mel* as a novel effector of the miR-34 regulatory network in stress. Although *Mel* has not previously been reported as a miR-34 target, its WD-repeat domain suggests a scaffolding or regulatory function in multiprotein complexes, raising the possibility that altered *Mel* expression could disrupt neuronal homeostasis or signaling under stress. Though we detected a modest increase in Mel levels in the head tissue of stressed flies compared to controls by Western analysis of stressed samples (Fig 1c-e), we were unable to detect an increase in its levels by proteomics. This could be due to a limitation in the number of technical replicates analyzed in proteomics, or to the fact that we were overexpressing Flag-Mel in only 5HT1B neurons, which number ~100. We also found that Western blot analysis only appeared to work when fresh samples were processed immediately, suggesting that the protein was unstable and that a small increase might be difficult to detect by proteomics. Nevertheless, peptides corresponding to Mel were detected in all the samples. Our proteomics analysis further confirms that stress and mel overexpression reprogram the *Drosophila* brain into a low-flux state characterized by suppression of nuclear output, intracellular trafficking, and synaptic remodeling, coupled with enhanced mitochondrial efficiency, detoxification, and structural stabilization, thereby preserving neuronal integrity.

## DISCUSSION

### miR-34 dosage tunes Stress Vulnerability and Resilience in *Drosophila*

In this study, we identified a conserved miR-34–*Mel* regulatory axis that modulates behavioral susceptibility to psychosocial stress in vivo. Previous studies indicate that *Drosophila* miR-34 is a critical modulator of brain aging and neurodegeneration(30). In the aging brain, miR-34 affects brain proteostasis by regulating its target *Lst8*, a subunit of the Tor Complex 1 (TORC1) (33). Using the genetically amenable *Drosophila*, we demonstrate that miR-34 regulates stress-induced depression-like behavior in a dosage-dependent manner through its direct target *melancholy (mel)*, a functional ortholog of mammalian *WDR26*. Importantly, genetic manipulation of this pathway selectively affects stress-induced behavioral states without altering baseline viability, indicating a specific role in stress responsiveness rather than generalized neural dysfunction.

We examined the effects of miR-34 overexpression using four different Gal4 drivers that drove expression of miR-34 pan-neuronally in adults (*3X ElavGS*), in the neurons specialized for learning and memory of adults (*MBGS*), in the serotonin-producing neurons (TrhGal4), and in serotonergic neurons expressing the 5-HT1B receptor (HT1BGal4) and found the effects to be most pronounced in 5-HT1B neurons. We found that this role of miR-34 is predominantly localized to serotonin-sensing cells, which are key postsynaptic targets of serotonergic input. Unlike Trh+ neurons, which broadly regulate neurotransmitter release, 5HT1B neurons gate the interpretation of serotonin signals within specific circuits. The enhanced behavioral effects observed upon miR-34 overexpression in 5-HT1B neurons likely reflect its role as a critical postsynaptic integrator of serotonergic signaling, in which modulation of signaling pathways can readily translate into circuit-level behavioral outputs with minimal compensatory buffering.

We also uncovered upstream regulators of *pri miR-34* under stress conditions. We found that downregulation of miR-34 in stress conditions is mediated by an ecdysone-driven transcriptional program that involves upregulation of *Broad-complex*. Knockdown of *Broad* prevents miR-34 downregulation and leads to stress resilience (Fig. 3). These findings position *Mel* as a previously unrecognized regulator of affective behavioral outcomes and establish miR-34 as a dosage-sensitive molecular link between psychosocial stress and persistent behavioral change.

### Epigenetic and genetic regulation of *WDR26* (WD Repeat Domain 26) in depression

Our functional analysis identified the Wdr26 ortholog Mel as the downstream effector of miR-34 in the context of stress-induced depression. Our analysis also showed that overexpression of *mel* is sufficient to induce a depression-like state in unstressed flies. The human ortholog of *Mel* (*CG7611*) is *WDR26*. *WDR26* encodes a seven WD-repeat-containing protein that functions as a scaffold protein to regulate several signaling pathways involved in stress responses, cell survival, and cell migration. One Genome-wide methylation study identified a differentially methylated probe in the WDR26 gene. This study, which examined epigenetic marks in monozygotic (MZ) twins that are discordant for depression, showed evidence that a CpG site within WDR26 is hypomethylated in the blood of individuals with a lifetime diagnosis of depression(77). Another meta-analytic study of genome-wide association data for MDD also suggested a role for the WDR26 rs11579964 single-nucleotide polymorphism in the etiology of depression (78). Expression of *WDR26* has also been proposed to be a peripheral blood biomarker for depressive disorders in animal models of depression (79). In addition, studies that have examined epigenetic marks in monozygotic (MZ) twins that are discordant for depression show evidence that a CpG site within WDR26 is hypomethylated in the blood of individuals with a lifetime diagnosis of depression(77). Given the existing information regarding the genetic and epigenetic regulation of *WDR 26* in depression, it is likely that its expression is fine-tuned at the post-transcriptional level by soon-to-be-discovered miRNAs. Using miRNA target prediction tools, we find that *WDR26* mRNA has target sites for other miRNAs but not for the miR-34 family (miR-142-5p, miR-29-3p, miR-218-5p, miR-302c-3p, miR-202-5p, miR-489-3p, miR-30-5p, miR-153-3p, miR-129-3p, miR-223-3p, miR-10-5p, miR-141-3p, miR-455-3p). Amongst the predicted miRNAs, miR-223-3p, miR-218-5p, miR-29-3p, miR-153-3p, miR-129-3p, miR-142-5p, and miR-10-5p have been shown to be involved in the pathogenesis, diagnosis, or treatment response of depression(11,18,20,80,81). To the best of our knowledge, our study is the first to demonstrate a direct role for Wdr26/Mel in depression-like behavior and provides causal functional validation in an animal model. Our analysis shows that overexpression of Mel in HT1B neurons is sufficient to induce behavioral deficits that are linked to stress-induced depression. We also show that *mel* knockdown in HT1B neurons confers resilience in stressed flies.

### Proteomics profiling revealed conserved pathways modulated in response to stress

Proteomic analysis of head tissue from control and stressed animals uncovered key pathways underlying stress-induced molecular remodeling. The chronic stress regime induced a coordinated downregulation of proteins spanning cytoskeletal organization, transcriptional and RNA regulatory machinery, metabolic pathways, calcium-dependent excitability, vesicular trafficking and secreted factors (Clusters 1 and 5), consistent with a systems-level shift in neuronal state. This pattern recapitulates a transition of stress-exposed neural circuits towards a low-plasticity, hypoactive neuronal state. Reduced abundance of actin-binding proteins likely limited synaptic remodeling, while suppression of the transcriptional machinery curtailed activity-dependent gene expression required for adaptation. Concurrent downregulation of metabolic enzymes indicates reduced energetic support, and decreased calcium signaling and vesicular trafficking that would likely impair neuronal excitability and neurotransmitter release, thus lowering synaptic output. Lastly, loss of secreted factors further indicates reduced intercellular communication. Although this coordinated response would initially serve to conserve energy and limit excitotoxicity, its persistence is likely to stabilize a maladaptive circuit function and promote depression-like behavioral deficits.

The categories of proteins that were upregulated upon stress but downregulated upon *mel* overexpression (Cluster 2) included chromatin regulators, transcriptional machinery, nuclear pore complex, RNA processing factors, protein translation and ribosome-associated factors, and signaling and trafficking systems. This cluster likely represents a stress-induced compensatory program that is actively suppressed when *mel* is elevated. Under chronic stress, the upregulation of chromatin modifiers, transcriptional machinery, RNA processing factors, and nuclear pore components suggests an attempt to reconfigure gene expression and enhance nuclear-cytoplasmic communication in response to sustained perturbation. This is consistent with a state of heightened transcriptional demand, where neurons are engaging epigenetic and transcriptional regulators to adapt to stress-induced signaling and maintain cellular homeostasis. Similarly, the increase in translation and vesicular trafficking components indicates a drive to support proteome remodeling and synaptic adjustment.

In contrast, the downregulation of these same pathways upon *mel* overexpression suggests that *mel* overexpression does not mimic the adaptive transcriptional activation phase of stress. Instead, mel accelerates the downstream maladaptive state in which neurons exhibit stress-like functional deficits without engaging compensatory gene expression programs. The suppression of chromatin, transcriptional, and translational machinery suggests a shift towards reduced nuclear output and proteostatic capacity, potentially locking neurons into a low-flexibility dysfunctional state. Thus, Cluster 2 likely reflects a set of proteins involved in an attempted adaptive response to stress, which is absent or actively inhibited upon *mel* overexpression. This distinction implies that while stress initially activates these pathways to cope with perturbation, *mel* bypasses this phase and directly induces a pathological endpoint, thereby recapitulating depression-like behavior without the intermediate compensatory response.

Our analysis also revealed Cluster 4 proteins that were upregulated under chronic stress but remained unchanged or slightly reduced upon mel overexpression, indicating that they represent a protective, homeostatic response program to stress that is not engaged upon *mel* overexpression. These proteins, linked to metabolism, proteostasis, vesicular trafficking, membrane remodeling, and redox balance, likely function to maintain cellular integrity and buffer stress-induced damage. The lack of induction despite *mel*-induced stress-like behavior suggests that *mel* drives a pathological state without activating compensatory defenses. This may leave neurons less capable of managing damage or adapting to perturbation. Thus, Cluster 4 represents a response program that is activated by the stress regime but is bypassed under *mel* overexpression, reinforcing the idea that *mel* selectively recapitulates maladaptive, rather than protective, aspects of the stress response. Thus, our proteomic analysis captured both canonical stress-response programs and distinct protein clusters that were either comodulated by stress and *mel* or divergently regulated, revealing that *mel* selectively recapitulates maladaptive stress-associated pathways while bypassing key compensatory and protective responses.

Taken together, this study reveals a conserved miRNA-34–scaffold protein (Mel) module that shapes behavioral responses to psychosocial stress, offering new insight into the molecular architecture of stress susceptibility.

## METHODS

### Animal (Rodent) Experiments and groups

All animal experiments were conducted in accordance with the guidelines of the Committee for the Purpose of Control and Supervision of Experiments on Animals (CPCSEA), Government of India, constituted under the Prevention of Cruelty to Animals Act, 1960. The study protocol was reviewed and approved by the Institutional Animal Ethics Committee (IAEC) of Shiv Nadar Institution of Eminence, which is registered with CPCSEA (SNIoE/IAEC/2024/1/014). All procedures involving animals adhered to CPCSEA guidelines for the care and use of laboratory animals, including provisions for housing, husbandry, and experimental procedures designed to minimize pain and distress. The principles of the 3Rs (Replacement, Reduction, and Refinement) were strictly followed throughout the study. 6-month-old Long-Evans rats were bred in the Centre for Integrative Research (CITRES) at the Shiv Nadar Institute of Eminence. Rats were housed in groups of three per cage, with free access to food and water, under a 14 h light/10 h dark cycle in a suitable environment with temperature (20–25°C) and humidity (40%–50%). Age-matched male rats were individually transferred to open cages a week before the experiment began to familiarize them with the environment. The rats were randomly divided into two groups (n=12 per group): the control group and the Chronic Unpredictable Mild Stress (CUMS) group. The CUMS method was used for establishing a depression-like state. After 3 weeks of exposure to CUMS regime, the behavioral analysis and molecular analysis were performed.

### Chronic Unpredictable Mild Stress (CUMS) Regime

6-month-old Long Evans rats were bred in the Center for Integrative Research (CITRES) at Shiv Nadar Institute of Eminence. They were fed a standard chow diet and maintained at a 14:10 Light-Dark cycle. The CUMS regimen was administered for 3 weeks. Every day, animals were exposed to two stressors, one in the morning and one at night. The regime was a modification of a previously published study, and the stressors are listed in Table S3(37). At the end of the 3-week regimen, behavioral assays were performed, following which the animals were first anesthetized using isofluorane, their blood was drawn through cardiac puncture, and they were euthanized using carbon dioxide overdose. Their brains were dissected in ice-cold PBS, and the hippocampus and PFC were carefully excised and stored in RNALater (Thermo Fisher Scientific, USA) at −20°C. RNA was extracted from this tissue using RNAiso Plus (Takara Bio, USA).

### Behavior tests in rodents

#### i. Body weight measurement:

The body weight of the rats in each group was measured at 6 P.M. each week using a weighing scale.

#### ii. Open Field Test (OFT):

Control and CUMS rats were introduced in a novel arena measuring 75cm×75cm (length × width) and recorded for 10 minutes. Their behavior was recorded using a webcam and tracked using AnyMaze Software. A central zone measuring 30 cm × 30 cm was defined in the software. The software recorded the rat's position and calculated movement parameters in the central and peripheral zones. After each rat, the arena was cleaned with 70% ethanol and patted dry before the next animal was placed inside.

#### iii. Marble burying test (MBT):

12 marbles were uniformly laid out in a novel cage containing a thick layer of bedding material. Animals were introduced to this novel cage and allowed to explore it for 30 minutes. A snapshot of the marbles' positions was taken before and after the test. After 30 minutes, the rats were returned to their home cage, and the number of marbles remaining on the surface was counted and recorded.

#### iv. Forced Swimming Test (FST):

Control and CUMS rats were gently released into a cylindrical swimming arena measuring 40cm in diameter and 50cm in height. Animals were recorded for 6 minutes, and the water was changed after every animal. After the assay was completed, the videos were analyzed for swimming time and immobility. Passive swimming without significant movement was scored as immobility. The rats were patted dry after the test and were allowed *ad libitum* access to food and water. The open field test and forced swimming test were not carried out on the same day.

### Fly strains and maintenance

Fly stocks were reared in standard cornmeal/agar medium at 24°C with a 12h light:12h dark cycle in 60% humidity. The cornmeal/agar food was prepared by mixing 86 g of cornmeal, 25g of sucrose, 51g of Dextrose, 15g of Yeast extract, 6g of agar, 1% Acid mix (10 ml), and Tegosept (5ml; 1 gm of Methyl 1195 4-hydroxybenzoate {SRL} in 5ml 100% Ethanol) per 1000 mL of food. The acid mix stock solution was prepared by combining propionic acid (164 ml milliQ water with 836 ml propionic acid {SRL}) and orthophosphoric acid (917 ml MilliQ water to 83 ml of orthophosphoric acid {SRL}). All experiments were performed in the *Drosophila* chamber Model DR-36VL (Percival Scientific, Inc, IA, USA). Detailed genotypes of all strains, as well as the sources of the genetic mutations and transgenes used in the study are listed in Table S1. Transgenesis (*UAS Flag CG7611*) was performed by the Fly Facility at Bangalore Life Science Cluster (CCAMP, Bengaluru, India). Stocks with multiple genetic elements were obtained by crosses. For steroid-mediated UAS-transgene control using the Gene-Switch driver, flies were fed a diet containing 200 μM RU-486 (Mifepristone, Cayman Chemicals, Ann Arbor MI). 5-HT1B Gal4 (BDSC 27637), Trh Gal4 (BDSC 38388), MB Gene Switch Gal4 (MBGS) (BDSC 81013), and 3xElav Gene Switch (3XElavGS) were used to overexpress and knock down genes and microRNAs. Virgin females from the Gal4 line were sorted and crossed to UAS driver lines. The F_1_ progeny flies were collected at 2-3 days of age. For the progenies of 5-HT1B Gal4 flies and Trh Gal4 flies, 2-3-day-old flies were directly used for the experiment. They were sorted by sex and maintained at a mean of 20 flies per standard cornmeal-yeast vial. They were then subjected to the variable stress regime. The progenies of 3x Elav Gene Switch flies and Mushroom Body Gene Switch flies were sorted according to sex and maintained in groups of 20. The genotypes of all the strains used in the study are indicated in the manuscript text and figure legends.

### Variable Stress Regime in *Drosophila* strains

Flies were anesthetized with CO_2_, after which they were sorted according to sex. 10 flies were placed in an empty vial, and heat shock was administered at 37°C in an incubator (39). Following that, male flies were allowed to fast in the same empty vial for 6 hours, while females were fasted for 8 hours. After the fasting period, flies were anesthetized and individually transferred to round-bottom plastic tubes containing food in their caps and were left at ambient light and temperature for 18 hours to ensure social isolation. After 18 hours of social isolation, flies were regrouped into groups of 10 in an empty vial and plunged into a −5°C bath for 10 minutes for males, while females were cold-shocked in 2 rounds of 10 minutes each. Flies were allowed to completely recover before performing any behavioral assays.

### Behavioral assays in *Drosophila* strains

#### i. Sucrose Preference Test (SPT):

Flies were fasted uniformly in empty vials for 1 hour before beginning the assay to develop an appetite for the liquids. The cotton plug was replaced with a sponge cap with two holes, into which 200 μL tips were cut and inserted. They were then offered a choice between 5% sucrose and water for 3 hours. 5% sucrose (SRL) and water were administered using 5 μL capillaries, which were inserted through the sponge caps. After 3 hours, the column of each liquid left after ingestion of flies was measured using vernier calipers. Sucrose intake and water intake per fly are calculated, and the PI is calculated. All vials were kept at 24°C and 50%RH.

Preference index (PI) = (Sucrose intake per fly − water intake per fly) / Total liquid intake per fly

#### ii. Forced Swimming Test (FST):

Flies housed individually in 14 ml polystyrene tubes were transferred to a novel arena containing 4 ml of 0.08% SDS, and their swimming times until absolute immobility were recorded. Each video was analyzed for latency to absolute immobility, and the time was recorded. After the test, flies were removed from the swimming arena and allowed to dry off to ensure their locomotory activity was not compromised. Flies that could not recover after the assay were eliminated from the analysis.

#### iii. Open Field Test (OFT):

Flies were sorted according to sex and housed in 14mL polystyrene tubes. They were then transferred to a novel arena after a 15-minute recovery and a 2-minute acclimatization. Their activity was then videotaped for 5 minutes and analyzed using open-access MATLAB-based software called DLTdv8(82). From the coordinates obtained per frame, the total distance covered during 5 mins of the assay was then calculated.

The same control panel *5HT1B Gal4> w^1118^* (unstressed and stressed flies) was used for all RNAi experiments (Figures 3G-L, 4J-P, 5F-K, S2B-G and S2I-N ). For clarity of reading, the panels have been segregated into different figures. The combined data is also presented as Fig S5.

### RNA extraction and Quantitative RT-PCR

Total RNA was extracted from dissected brain tissue, head, and Kc167 cells using RNAiso Plus (Takara Bio, Inc). Fly tissues, or cells, were homogenized in 0.5 ml of RNAiso plus, and 50mg of rat hippocampus was homogenized in 1ml of RNAiso plus using a bead ruptor before extraction. cDNA was synthesized using High-Capacity cDNA Reverse Transcription kit (Thermo Fisher Scientific, USA) and Bio-Rad T100 Thermal Cycler. The cDNA was used as a template for quantitative real-time PCR using Taqman MicroRNA assay kits (Thermo Fisher Scientific, USA) for specific miRNAs (miR-34, miR-124, let-7, sno442, and Synthetic *C. elegans* miR-39) or Takara SYBR Premix Ex-Taq plus and analyzed on the Biorad CFX Duet Real-Time PCR machine as described in our previously published studies(39,83). The expression of microRNAs was normalized to sno442 or synthetic-Cel-39, while the expression of genes was normalized to Actin-5c. For the RT-PCR done in Figure 1J, the RT-PCR involved poly-A tailing at 37°C followed by annealing if an Oligo dT-URP adaptor by incubating at 60°C for 10 minutes followed by 22°C for 5 minutes. Reverse transcription was performed with M-MuLV RT (NEB), and Real-time qPCR was performed with KAPA SYBR FAST qPCR Master mix.

### Plasmids and transgenes

The wild type and mutant Luciferase reporter constructs were generated by annealing the oligo pairs 632/633 and 634/635, respectively, and cloning into the *NotI*-*XhoI* site of pSicheck 3 vector. The N-terminal Flag-tagged *CG7611* cDNA was sub-cloned as an *EcoRI-XbaI* fragment into pUAST attB using the primers 644 and 645 by using the LP01609 plasmid (DGRC). The *mir-34* primary transcript was generated by annealing the oligo pair 638/639 and cloned into the *EcoRI-XhoI* site of pUASTattB. All PCRs were performed with High-fidelity Phusion enzyme (Thermo Fisher Scientific, USA), and all clones were verified by sequencing. The primers used for cloning are listed in Table S2.

### Cell culture and Luciferase Reporter Assay

Kc167 cells were cultured in T75 flasks at a density of 1.5 × 10^6^ cells/ml in M3+BPYE+5% FBS medium. At around passage 7-8, they were transferred to CCM3 medium. Cells were maintained at 25°C, 50% RH. Kc167 cells were transfected using 300ng of Tub Gal4, pUASt attB vector or pri-miR-34 pUASTattB, and predicted miR-34 binding site or its mutant cloned in psicheck 3. Transfection was performed using Lipofectamine 3000 (Thermo Fisher Scientific, USA) according to the manufacturer's protocol. Briefly, DNAs were mixed with P3000, and lipofectamine was mixed with CCM3 medium and incubated for 5 minutes; both were then combined and incubated for 20 minutes to allow complex formation. This complex was then added to Kc167 cells seeded in a 48-well plate, and transfection was allowed to proceed for 96 hours. After 72 hours, the cells were transferred to 1.5 ml MCTs (Eppendorf) and centrifuged at 2500 rpm for 5 minutes in an Eppendorf 5427R centrifuge. The media was removed and cells were washed in 1x PBS. Cells were then resuspended in chilled 1x PLB buffer (Promega Corporation) and incubated on ice for 20 minutes, after which they were stored in −80°C.

Dual Luciferase Assay was performed using the Promega Dual Luciferase Assay System (Promega Corporation). Cells were thawed and vortexed for 15 minutes to lyse them. They were then centrifuged at 2500 rpm for 5 minutes. 30μL of the supernatant was then pipetted into 96-well transparent plates (Applied Biosystems), and an equal volume of LAR II reagent was added. Luminescence was measured at the kinetic endpoint of 1000ms using Vector Nivo multimode plate reader (Revvity). Subsequently, 30μL of Stop-and-Glo reagent was added, and endpoint luminescence was recorded at 1000ms. Renilla luminescence was normalized to Firefly luminescence, and fold repression was calculated.

### Western Blotting

Flies were anesthetized, their heads were rapidly cut, and the heads were placed in a 1.5ml MCT containing 100 μL chilled RIPA buffer. The heads were homogenized on ice using bead ruptor and the lysate was centrifuged at max speed for 20 minutes at 4°C. Supernatant was transferred to a new tube and stored at −80°C. Protein concentration was measured using Biorad Protein Estimation Reagent (Bio Rad, USA) as per the manufacturer’s protocol. Lysates were used for western blot analysis using 1:1500 anti-tubulin antibody, 1:1500 anti-CG7611 antibody (Boster Bio). Primary antibodies were detected using HRP-conjugated 1:2500 antimouse antibody. The chemiluminescence signal was detected using Biorad Chemidoc Touch Image Gel Imaging System (Bio Rad, USA).

### Immunofluorescence

Adult *Drosophila* brains were dissected in PBS, and Immunofluorescence was performed as described previously (83). Tissues were fixed in 4% paraformaldehyde for 30 minutes and washed three times for 5 min each in 1X PBS +0.3% Triton X-100. Primary antibodies included a mouse anti-Flag antibody (Sigma-Aldrich). After incubation with the secondary antibody, tissues were washed 3 times for 5 minutes, stained with DAPI (1:100), and mounted. Slides were analyzed under a Nikon Ti2E, AXR NSPARC. Confocal stacks were merged using NIS-Elements software.

### Proteomic analysis

Age-matched control, stressed flies, or *Mel* overexpressing flies were used for lysate preparation. Protein lysate was prepared by freezing and homogenizing fly head tissue (n=30-40 heads/replicate) in PhosphoSafe extraction agent (Sigma-Aldrich) buffer (100ul) supplemented with 1X protease inhibitor complex and 0.1% nuclease enzyme. The samples were centrifuged for 15 min at maximum speed at 4°C in a microfuge, and the supernatant was collected, quantified, and processed for Proteomic analysis as described in a previous study(84).

Protein lysates were mixed with UA buffer (50mM Tris, 75mM NaCl, and 8mM Urea) in a filter tube (Millipore UFC500396) to a volume of 400 μL. The filter was centrifuged at 12000g for 20 minutes at 4°C, and the flow-through was discarded from the collection tube. Another 400 μL of UA buffer was added to the filter tube, and the tube was centrifuged two more times. 300 μL of UA buffer and 6 μL of 1 M DTT buffer were added to the filter tube, which was then incubated at 37°C for 1 h. After incubation, the tube was cooled to 25°C, and 30 μL of 1 M IAA/Alkylation buffer (1 M Iodoacetamide in UA buffer) was added to the filter. The filter tube was centrifuged at 12000g for 20 minutes at 4°C, and the flow-through was discarded from the collection tube, followed by the addition of 400 μL of UA buffer. This step was repeated two more times, followed by the addition of 400 μL of ABC buffer (50mM Ammonium bicarbonate) to the filter tube. The filter tube was centrifuged at 12000g for 20 minutes at 4°C, and the flow-through was discarded from the collection tube. Then, 200 μL of ABC buffer with Trypsin (enzyme-to-protein ratio 1:50) was added, and the tube was incubated at 37°C for 20 h after the collection tube was changed. After the incubation, the filter unit was centrifuged for 12000g for 20 minutes at 4°C, followed by the addition of 200 μL of ABC buffer and another centrifugation step. The tryptic peptide eluates were collected, dried in a SpeedVac vacuum dryer, and reconstituted in 0.1% Formic acid (FA). The reconstituted peptides were cleaned and desalted using a C18 ZipTip (Thermo Fisher Scientific). Samples were eluted from the cartridge in 2% acetonitrile, 0.1% formic acid, followed by LC/MS.

### Mass spectrometry data processing and statistical analysis

Raw peptide-level quantitative data generated using DIA-NN were imported into R (v4.3), and intensity columns corresponding to individual MS runs(85). A sample annotation table was constructed by parsing run names, assigning samples to Stress, Control, or OE conditions, and retaining selected biological replicates. Peptide-level data were merged with annotations, and entries with missing or zero intensities were removed prior to downstream analysis.

Data were processed using the MSstats framework following established DIA workflows (86,87). Prior to normalization, log_2_-transformed intensity distributions were inspected to assess data quality and detect outliers. Normalization was performed using median equalization, and protein-level summarization was carried out using Tukey’s median polish (TMP). Missing values were handled using model-based imputation. Normalized protein intensities were aggregated across replicates to generate a protein abundance matrix, which was scaled and subjected to principal component analysis (PCA) to evaluate global proteomic variation and sample clustering. Differential protein abundance was assessed using linear mixed-effects models implemented in MSstats (86,87). A contrast matrix was defined to evaluate pairwise comparisons (Stress vs OE, Control vs Stress, and Control vs OE). Proteins with an adjusted *P* < 0.05 and ∣log_2_ fold change∣ > 0.58 (~1.5-fold) were considered significantly differentially abundant, and results were visualized using volcano plots.

Significant proteins were further analyzed using k-means clustering (up to 5 clusters), and the resulting expression patterns were visualized as heatmaps of scaled intensities annotated by condition.

### Statistical Analysis

Statistical analysis and data presentation was performed using GraphPad Prism 11 software and/or Microsoft Excel. Other details of statistical analysis can be found in the figure legends. Statistical significance was set at p <0.05.

### Language Editing and Refining

ChatGPT was used to restructure some parts of the Introduction and Discussion section to improve clarity and readability. All AI-generated suggestions were critically reviewed, edited and verified by the authors for scientific accuracy. The authors take full responsibility for the final content of the manuscript.

## Supplementary Material

Supplement 1

This is a list of supplementary files associated with this preprint. Click to download.

TableS6.xlsx

TableS4.xlsx

TableS5.xlsx
