# Supplementary material for: miR-34 regulates stress-induced depression-like state through the WDR26 ortholog melancholy in Drosophila
Source: Res Sq. 2026 Apr 8:rs.3.rs-9339604. Preprint. [Version 1] doi: 10.21203/rs.3.rs-9339604/v1 (PMC13082156; doi:10.21203/rs.3.rs-9339604/v1)
Supplement: Supplement 1 [file NIHPPrs9339604v1-supplement-1.pdf]

Supplementary data

Table S1. *Drosophila melanogaster* strains used in the study.

Stock	Genotype	Source/ No.	Figure
UAS pri miR-34	w^{1118}; *P{y[+t7.7] w[+mC]=UAS-LUC-mir-34.T}attP2*	BDSC 41158	4B-H, 4J-P
5-HT1B-GAL4	*w[*]; P{w[+mC]=5-HT1B-GAL4.Y}3*	BDSC 27637	3G-L, 4J-P, 6F-M,7A-I, S2B-G, S2I-N, S5A-F
Trhn-GAL4	w^{1118}; *P{w[+mC]=Trhn-GAL4.long}2*	BDSC 38388	S1B-G
MB-GS	w^{1118}; *P{w[+mC]=MB-Switch}3/TM6C, Sb[1]*	BDSC 81013	S1I-N
UAS CG7611^{RNAi}	*y[1] sc[*] v[1] sev[21]; P{y[+t7.7] v[+t1.8]=TRiP.HMS05669}attP40/CyO*	BDSC 67776	6F-M, S5A-F
UAS Su(z)12 ^{RNAi}	*y[1] v[1]; P{y[+t7.7] v[+t1.8]=TRiP.JF01706}attP2*	BDSC 31191	S2B-G, S5A-F
UAS Pcl^{RNAi}	*y[1] sc[*] v[1] sev[21]; P{y[+t7.7] v[+t1.8]=TRiP.HMS00897}attP2/TM3, Sb[1]*	BDSC 33946	S2I-N, S5A-F
UAS Nrx IV^{RNAi}	*y[1] sc[*] v[1] sev[21]; P{y[+t7.7] v[+t1.8] =TRiP.HMS01991}attP40*	BDSC 39071	S5A-F
UAS Gw^{RNAi}	*y[1] sc[*] v[1] sev[21]; P{y[+t7.7] v[+t1.8]= TRiP.HMS01638}attP40*	BDSC 37496	S5A-F
WT for miR-34^{KO}	w^{1118}	BDSC 5905	5A-H
miR-34^{KO}	*miR-34KO/sb*	Nan Liu/Nancy Bonini	5A-H
Wild type	*Canton-S*	BDSC	2C-L,6A-E
UAS Flag CG7611	w^{1118};*P{w+, UAS-Flag CG7611::SV40}attP40/ CyO*	This study	7A-I,S3A-H
Balancer	*w[1118]/Dp(1;Y)y[+]; CyO/nub[1] Adc[b-1] sna[Sco] lt[1] stw[3]; MKRS/TM6B, Tb[1]*	BDSC 3703	7A-I, S3A-H
3X ElavGS	*P{elav-Switch.O}GS −1A; P{elav-Switch.O}GS-3A, P{elav-Switch.O} GSG301*	Scott Pletcher	4B-H,S3A-H
UAS Br-c^{RNAi}	y[1] v[1]; P{y[+t7.7] v[+t1.8]=TRiP.HMS00042} attP2/TM3, Sb[1]	33641	3G-L

Table S2. Primers used in the study.

Description	Sequence	Lab #	Source	Figure
Br-c QRT PCR For	CCTTCGTGGATGTGACCCTC	GC603	This study	3A-B,D
Br-c QRT PCR Rev	TTTGCAGGGTGTGCTCTTGA	GC604	This study	3A-B, D
CG7611 QRT PCR For	TTTCGGGCGGACAACAAAAC	GC601	This study	6A-B, 6P-Q, 6L-M
CG7611 QRT PCR Rev	TGAACGCGGGCTATCAAAGT	GC602	This study	6A-B, 6P-Q, 6L-M
dme Actin QRT For	CACACCAAATCTTACAAAATGTGT	GC 23	This study	3A-B, 3D-E, 6A-B, 6P-Q, 6L-M
dme Actin QRT Rev	AATCCGGCCTTGCACATG	GC 24	This study	3A-B, 3D-E, 6A-B, 6P-Q, 6L-M
dme pri miR-34 RTPCR For	GCTATGCGCTTTGGCAGTG	GC605	This study	3E-F
dme pri miR-34 RTPCR Rev	GACGAGATTCCAGACAATCCGA	GC606	This study	3E-F
Luciferase wt CG7611 For	GGCCGCTCGTCGTTGCTACAAACTGCCAT	GC632	This study	6O
Luciferase wt CG7611 Rev	TCGAGATGGCAGTTTGTAGCAACGACGA	GC633	This study	6O
Luciferase mut CG7611 For	GGCCGCTCGTCGTTCGATCAATGACGGATc	GC634	This study	6O
Luciferase mut CG7611 Rev	TCGAGgATCCGTCATTGATCGAACGACGAgc	GC635		6O
EcoRI UAS pri miR-34 For	AATTCGCTATGCGCTTTGGCAGTGTGGTTAGCTGGTTGTGTAGCCAATTATTGCCGTTGACAATTCACAGCCACTATCTTCACTGCCGCCGCGACAAGCc	GC638	This study	6O
Xho1 pri miR-34 Rev	TCGAGGCTTGTCGCGGCGGCAGTGAAGATAGTGGCTGTGAATTGTCAACGGCAATAATTGGCTACACAACCAGCTAACCACACTGCCAAAGCGCATAGCg	GC639	This study	6O
EcoR1 3X Flag-CG7611 For	CGGAATTCCCATGGACTACAAAGACCATGACGGTGATTATAAAGATCATGACATCGATTACAAGGATGACGATGACAAGCAGAGCACCAGTTCCACGTCG	GC644	This study	6A, 7A-I, S3A-H
XbaI CG7611 Rev	GATCTAGTTAAGTCATATTCCAGGAGGACG	GC645	This study	6A, 7A-I, S3A-H

CG7611 Colony PCR Rev	CGGTGATCGTTGCCAATTTC	GC646	This study	6A, 7A-I, S3A-H
Rat PSMB9 QRTPCR For	GTGTCGTGGTGGGCTCTGATTC	GC692	This study	1I
Rat PSMB9 QRTPCR Rev	GTCCGCTATGGCTTGGGCATC	GC 693	This study	1I
Rat GAPDH QRTPCR For	CAACTCCCTCAAGATTGTCAGCAA	GC 694	This study	1I
Rat GAPDH QRTPCR Rev	GGCATGGACTGTGGTCATGA	GC 695	This study	1I

Table S3. CUMS regime for Long Evans Rats.

	Day 1	Day 2	Day 3	Day 4	Day 5	Day 6	Day 7
**Week 1**	Restraint (4h)<br>Stroboscopic lighting (overnight)	Water deprivation (24h)<br>Overnight illumination	Food deprivation (24h)<br>Tom cat spray (10 mins)	Cage tilt (24h)<br>Day-Night reversal	Restraint (4h)<br>Water deprivation (24h)	Water deprivation (continued)<br>Wet bedding (24h)	Wet bedding (continued)<br>Food deprivation (24h)
**Week 2**	Food deprivation (continued)<br>Stroboscopic lighting	Tom cat spray (10 mins)<br>Restraint (4h)	Water deprivation (24h)<br>Day-Night reversal	Cage tilt (24h)<br>Wet bedding (24h)	Wet bedding (continued)<br>Food deprivation (24h)	Food deprivation (continued)<br>Restraint (4h)	Water deprivation (24h)<br>Overnight illumination
**Week 3**	Cage tilt (24h)<br>Stroboscopic lighting	Restraint (4h)<br>Food deprivation (24h)	Food deprivation (continued<br>Wet bedding (24 hours)	Wet bedding (continued)<br>Tom cat spray (10 mins)	Restraint (4h)<br>Day-night reversal	Water deprivation (24h)<br>Cage tilt (24 hours)	Cage tilt (continued)<br>Stroboscopic lighting (overnight)

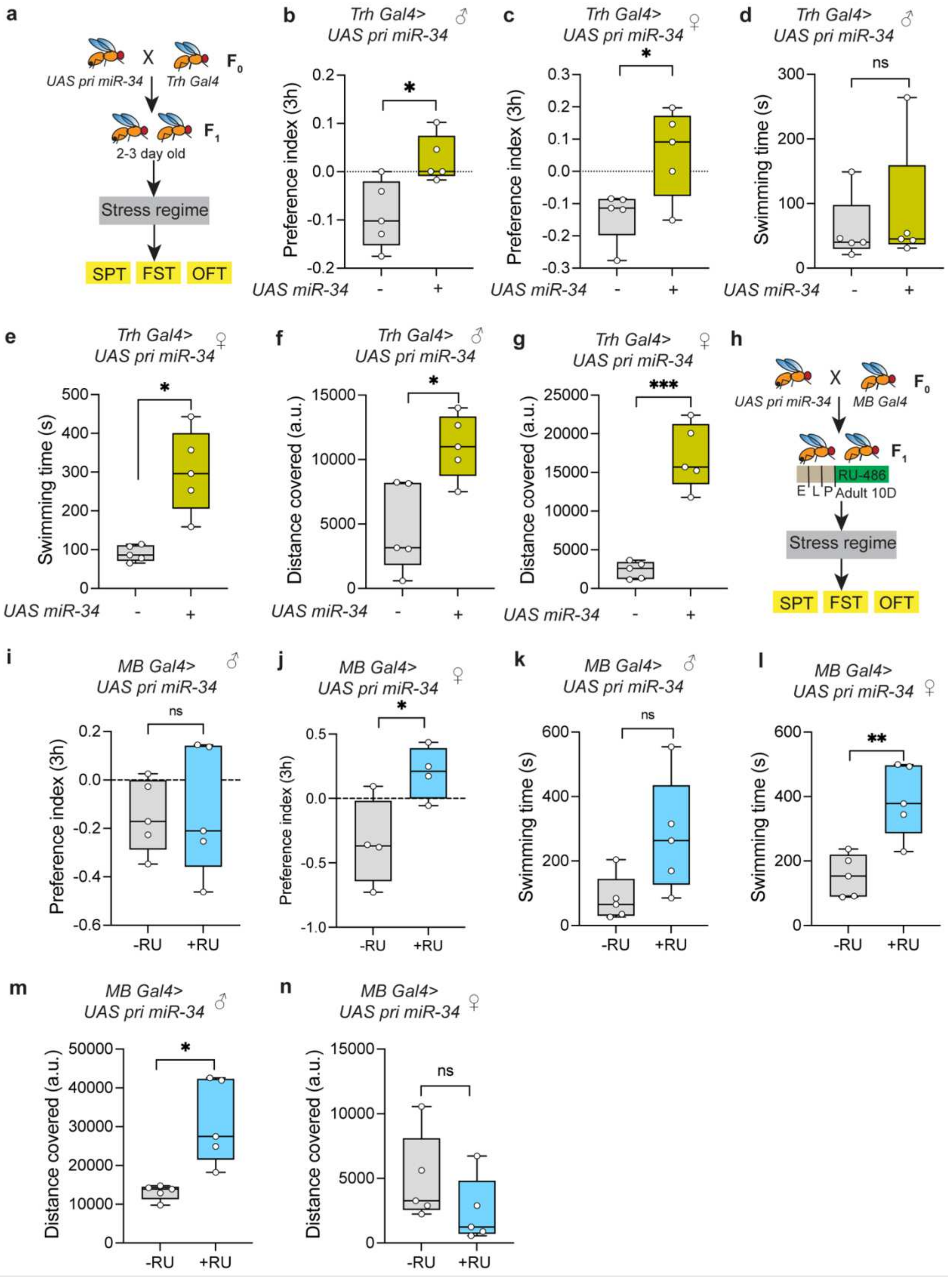

8 **Figure S1. MiR-34 overexpression in Tyrosine hydroxylase and Mushroom Body neurons**

differentially modulates stress-induced behavioral outcomes. (a) Schematic of the genetic cross and workflow for Trh-Gal4–driven miR-34 overexpression. F_1 progeny (2–3 days old) were subjected to a stress paradigm followed by the SPT, FST, anOFT). **(b–c)** Sucrose preference indices in stressed male (b) and female (c) *Trh>UAS-pri miR-34* flies, showing increased sucrose preference upon miR-34 overexpression in both sexes. **(d–e)** Forced swim test performance in stressed male (d) and female (e) flies, reflecting helplessness-like behavior; miR-34 overexpression increased swimming time in females but not in males. **(f–g)** Open field test showing exploratory behaviour in males (f) and females (g), with miR-34 overexpression enhancing exploratory activity in both sexes. **(h)** Schematic of the MBGS virgin females crossed to *UAS pri miR-34* males for adult-specific miR-34 overexpression. **(i–j)** Sucrose preference indices in stressed male (i) and female (j) *MB Gs4>UAS-pri miR-34* flies with (+RU) or without (–RU) RU486 induction, showing relief from anhedonia in RU-induced females but not males. **(k–l)** Forced swim test in stressed males (k) and females (l), revealing increased swimming time only in RU-induced females. **(m–n)** Distance traveled by stressed males (m) and females (n) in an open field arena, with RU-486-induced miR-34 expression enhancing exploratory activity in males but not females. Data are presented as box plots with individual data points overlaid, with whiskers extending from the minimum to the maximum. Statistical comparisons were performed using unpaired t-tests with Welch's correction. ns, not significant; $*p < 0.05$, $**p < 0.01$, $***p < 0.005$. Genotypes of strains used in this figure: **(b-g)** *TrhGal4>UAS pri miR-34: P{w[+mC]=Trhn-GAL4.long}2/+;P{y[+t7.7] w[+mC]=UAS-LUC-mir-34.T}attP2/+;* **(i-n)** *MBGS>UAS pri miR-34: P{w[+mC]=MB-Switch}3/P{y[+t7.7] w[+mC]=UAS-LUC-mir-34.T}attP2.*

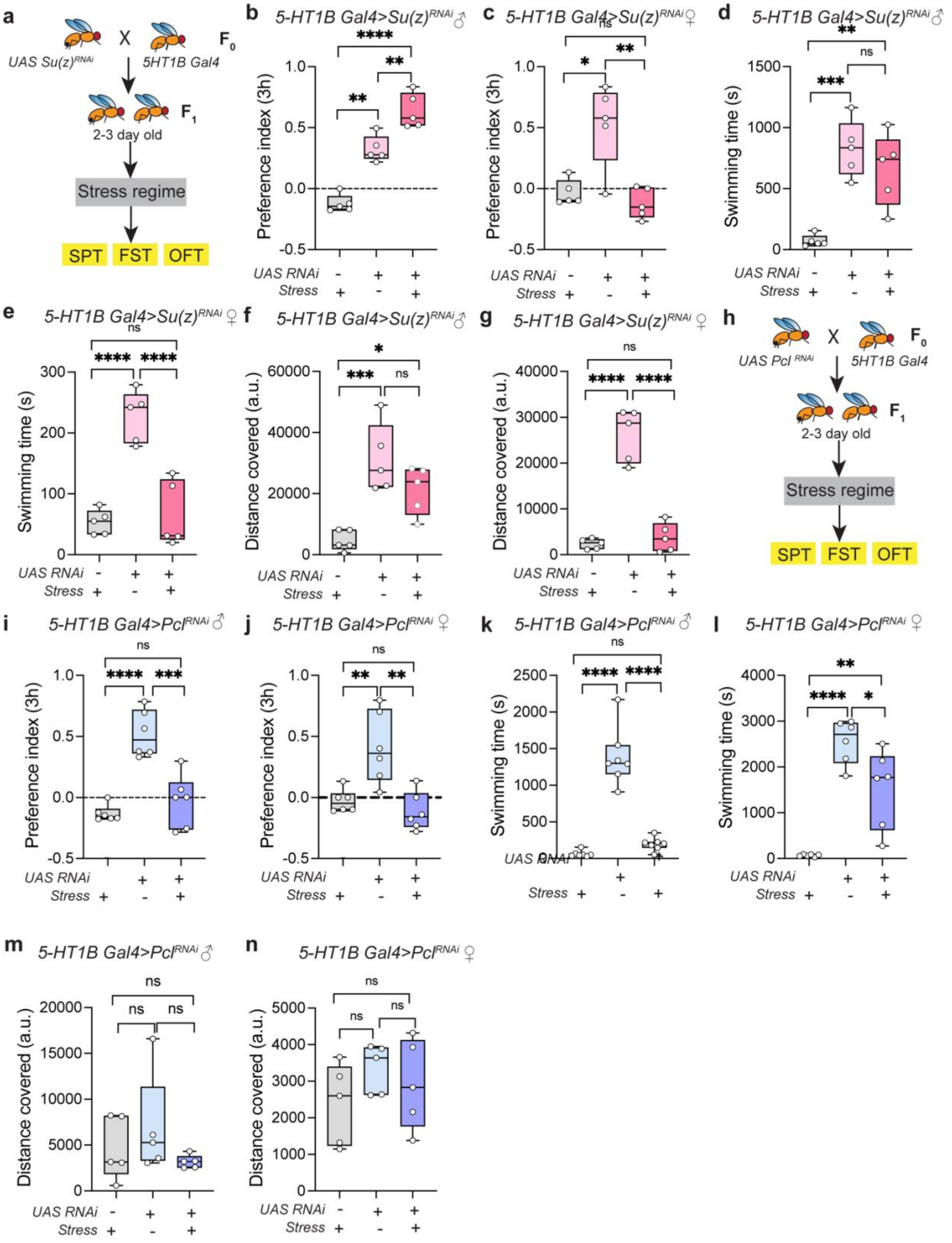

Figure S2. Knockdown of *Su(z)* and *Pcl* in 5-HT1B neurons modulates stress-

induced behavioral responses in a sex-specific manner. (a) Schematic showing genetic crosses used to generate F_1 progeny expressing *Su(z)*^{RNAi} under 5-HT1B-Gal4 control, followed by exposure to stress paradigm and behavioral assays. **(b–g)** Behavioral outcomes of Su(z) knockdown. **(b–c)** SPT in stressed males (b) and females (c) showing increased sucrose preference in *5-HT1B Gal4>UAS Su(z)*^{RNAi} males but no significant effect in females. **(d–e)** Forced swim test (FST) in stressed males (d) and females (e) revealing increased swimming time upon Su(z) knockdown selectively in males. **(f–g)** OFT in stressed males (f) and females (g) showing increased exploration in males but not females following Su(z) knockdown. **(h–n)** Behavioral outcomes of knockdown of *Polycomblike* (*Pcl*). (h) Genetic crosses set to knock down *Pcl* in 5-HT1B neurons. **(i–j)** SPT in stressed males (i) and females (j), both showing increased sucrose preference upon knockdown of *Pcl*. **(k–l)** FST in males (k) and females (l) demonstrates increased swimming time in both sexes following Pc knockdown. **(m–n)** OFT in males (m) and females (n) showed no significant change in exploratory activity after knockdown of *Pcl*. Data are shown as box-and-whisker plots with individual points overlaid. Statistical comparisons were performed using one-way ANOVA with Bonferroni correction. ns, not significant; *$p < 0.05$, **$p < 0.01$, ***$p < 0.005$, ****$p < 0.001$. Genotypes used in this figure **(b-g)** *y[1] v[1]; P{y[+t7.7] v[+t1.8]=TRiP.JF01706}attP2/ P{w[+mC]=5-HT1B-GAL4.Y}3,* **(i-n)** *y[1] sc[*] v[1] sev[21]; P{y[+t7.7] v[+t1.8]=TRiP.HMS00897}attP2/TM3, Sb[1]/+; P{w[+mC]=5-HT1B-GAL4.Y}3/+*

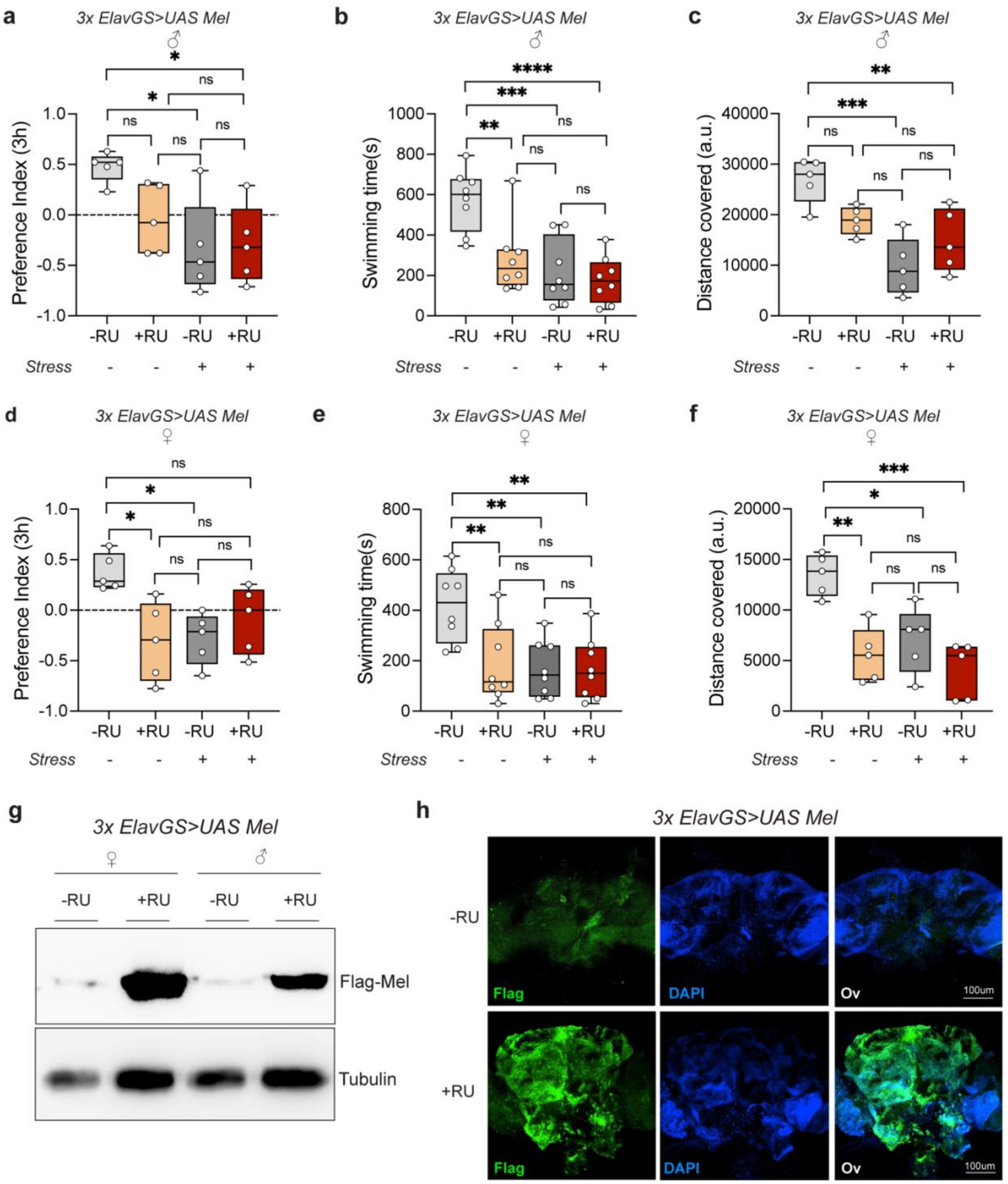

Figure S3. Pan-neuronal overexpression of *Mel* mimics behavioral signatures of chronic stress and phenocopies stress-induced deficits under basal conditions. (A–F) Behavioral assessment of male (A–C) and female (D–F) *3xElavGS>UAS-Mel* flies following treatment with

(+RU) or without (–RU) RU-486, and exposure to non-stress (–) or stress (+) conditions. **(A, D)** PI over 3 h for male (A) and female (D) flies. Induction of *CG7611* (+ RU) significantly reduces sucrose preference compared to basal controls (–/–), mimicking the anhedonia-like behavior seen in stressed controls (–/+), with no significant additive effect in the stress + overexpression group. **(B, E)** Total swimming time in male (B) and female (E) flies during the forced swim test (FST). A significant reduction in swimming duration is observed in +RU/– flies, indicating that *Mel* overexpression is sufficient to induce behavioral despair similar to environmental stress. **(C, F)** Exploratory behavior quantified by total distance traveled in the open field test (OFT) for male (C) and female (F) flies. Exploratory activity is significantly diminished upon either *CG7611* induction or stress exposure. **(G)** Western Blot analysis of Mel levels upon overexpression throughout all neurons using the *3xElavGS* driver. *3xElavGS>UAS* Mel flies were fed RU-486-supplemented food for 5 days, and heads were cut for protein isolation and probed with anti-Flag antibody. Male and female flies fed RU-486-containing food show a significant increase in Flag-Mel levels, while protein expression was found to be negligible in vehicle-fed flies (-RU). **(H)** Representative confocal images of adult *Drosophila* brains validating the Mel overexpression driven by *3xElavGS* driver in the presence of RU-486, stained using DAPI and anti-CG7611 antibody (green). The box plot shows the means of each group, with the box spanning from the first to the third quartile and whiskers extending from the minimum to the maximum data points, with individual data points overlaid. Statistical comparisons were made using one-way ANOVA with Bonferroni’s correction. ns, not significant; *$p < 0.05$, **$p < 0.01$, ***$p < 0.001$, ****$p < 0.0001$. Genotypes used in this figure: **(A-H)** *ElavGS > UAS Flag mel: P{elav-Switch.O}GS−1A/+;P{w+,UAS-Flag mel}attP40/+;P{elav-Switch.O}GS-3A, P{elav-Switch.O} GSG301/P{w+, UAS-Flag chin::SV40} attP2 / +.*

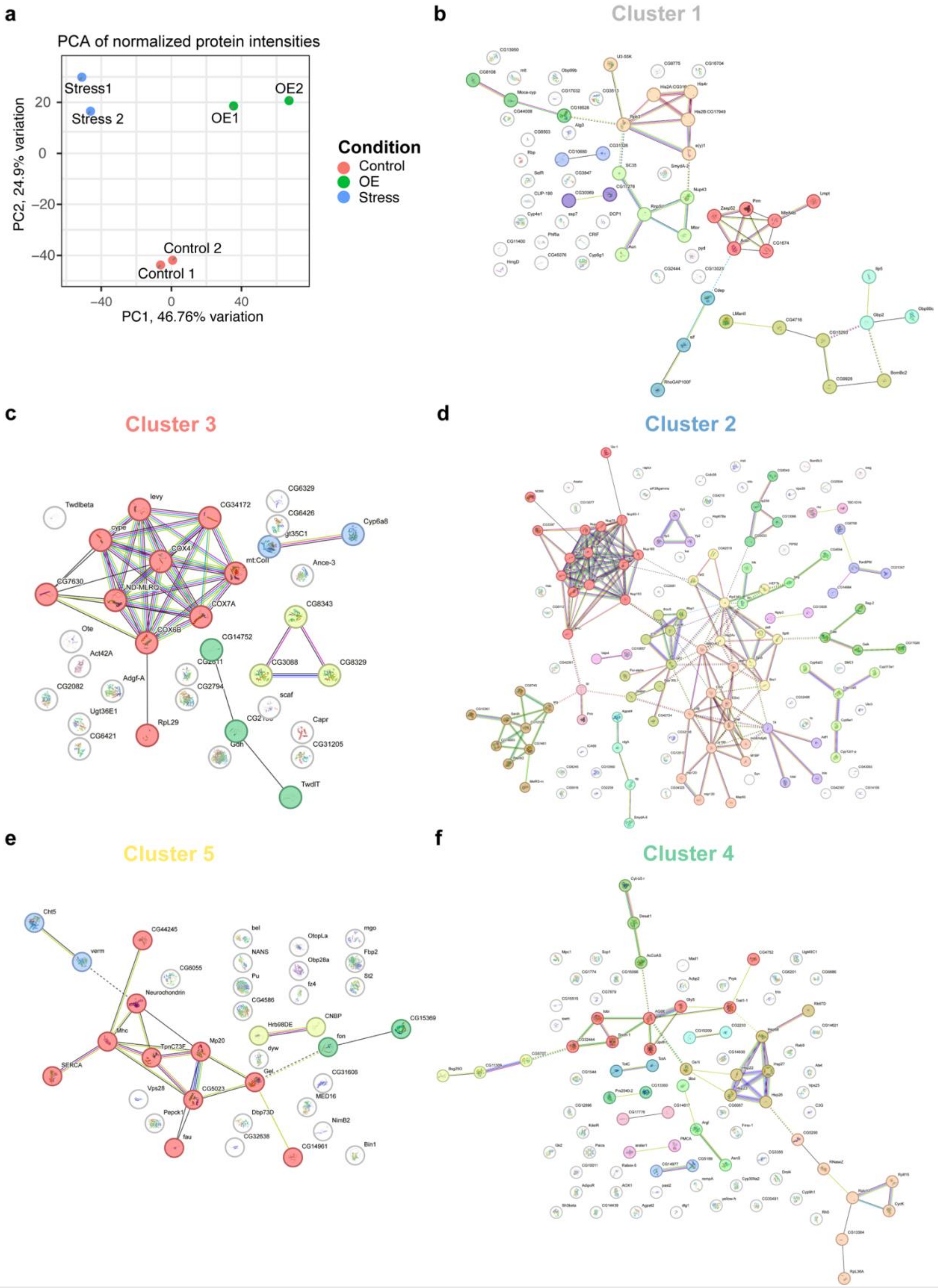

Figure S4. Proteomic profiling and interaction networks under different conditions. (A)

Principal component analysis (PCA) of normalized protein intensities showing separation of samples from control, overexpression (OE), and stress conditions. **(B–F)** Protein–protein interaction networks of groups first identified by K-means clustering of significantly differentially regulated proteins generated using the STRING database and clustered using the Markov Cluster (MCL) algorithm with an inflation parameter of 2 to identify densely connected modules (1). Each uniquely colored group of connected nodes represents a closely connected sub-cluster of proteins associated with specific functional modules in the dataset.

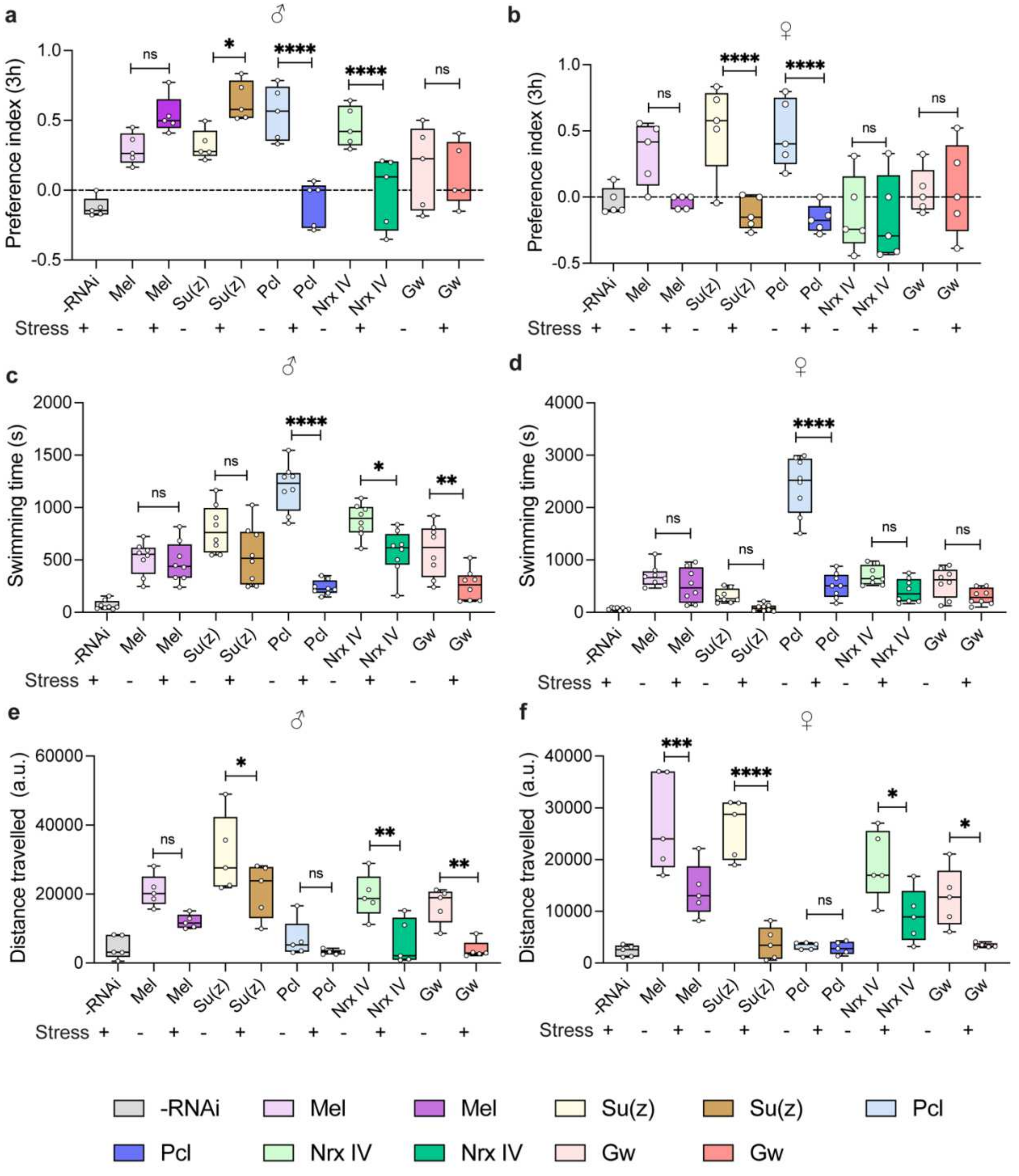

Figure S5. *CG7611/Mel* is a functionally relevant target of miR-34, which regulates behavioral outcomes. Five predicted miR-34 targets, namely *Mel, Su(z), Pcl, Nrx IV,* and *Gw,* were knocked down in 5-HT1B neurons, and behavior was assessed after exposure to a stress

regimen. **(a-b)** PI of flies measured at 3 hours in males **(a)** and females **(b)**. In males (a), *mel* knockdown does not alter the PI after exposure to a stress regimen compared with unstressed flies of the same genotype, indicating that anhedonia is alleviated. Su(z) knockdown leads to significant improvement in sucrose preference, while *Pcl* and *Nrx IV* knockdown do not improve PI post stress exposure, as it is significantly decreased in comparison to unstressed flies, and *G9a* (*G9a*) knockdown does not affect sucrose preference. In females (b), RNAi-mediated knockdown of *Mel*, *Nrx-IV*, and *Gw* does not lead to significant changes in preference indices upon stress or upon *Su(z)* and *Pcl* knockdown. Sucrose preference significantly reduces after being subjected to a stress regime compared to unstressed controls of the same genotype. **(c-d)** Swimming activity of flies measured in FST upon knockdown of miR-34 targets in males (c) and females (d). Swimming time exhibited by male flies upon knockdown of *Mel* and *Su(z)* does not differ significantly from their unstressed controls, indicating relief from depression-associated despair, while *Pcl*, *Nrx IV,* and *G9a* knockdown significantly enhanced despair-like behavior exhibited by reduced swimming time. (d) Swimming time upon *Mel*, *Su(z)*, *Nrx Iv,* and *G9a* knockdown remains unchanged upon stress exposure in comparison to unstressed controls, while a significant reduction is observed upon Pcl knockdown in comparison to its unstressed control. **(e-f)** Exploratory activity of male and female flies upon knockdown of miR-34 targets in 5-HT1B neurons. (e) In males, *Mel* and *Pcl* knockdown does not alter exploratory locomotion, as it remains comparable to unstressed controls; however, upon knockdown of *Su(z)*, *Nrx IV,* and *G9a*, exploratory activity decreased significantly in stressed flies. (f) In females, exploratory activity decreases upon stress exposure in comparison to controls upon *Mel*, *Su(z)*, *Nrx IV,* and *G9a* knockdown, while it remains unchanged upon *Pcl* knockdown. The box plot shows the means of each group, with the box spanning from the first to the third quartile and whiskers extending from the minimum to the maximum data points, with individual data points overlaid. Statistical comparisons were made using one-way ANOVA with Bonferroni's correction. ns, not significant; *p < 0.05, **p < 0.01, ***p < 0.001, ****p < 0.0001. (A-F) Genotypes of strains used: *y[1] v[1];*

P{y[+t7.7] v[+t1.8]=TRiP.JF01706}attP2/ P{w[+mC]=5-HT1B-GAL4.Y}3, y[1] sc[*] v[1] sev[21]; P{y[+t7.7] v[+t1.8]=TRiP.HMS00897}attP2/TM3, Sb[1]/+; P{w[+mC]=5-HT1B-GAL4.Y} 3/+, y[1] sc[*] v[1] sev[21]; y[1] sc[*] v[1] sev[21]; P{y[+t7.7] v[+t1.8]= TRiP.HMS01638} attP40/+; P{w[+mC]=5-HT1B-GAL4.Y}3/+, +/+; P{w[+mC]=5-HT1B-GAL4.Y}3/+,

References

1. Szklarczyk, D., Kirsch, R., Koutrouli, M., Nastou, K., Mehryary, F., Hachilif, R., Gable, A.L., Fang, T., Doncheva, N.T., Pyysalo, S. *et al.* (2023) The STRING database in 2023: protein-protein association networks and functional enrichment analyses for any sequenced genome of interest. *Nucleic Acids Res*, **51**, D638–d646.
